# Graph-Based 3-Dimensional Spatial Gene Neighborhood Networks of Single Cells in Gels and Tissues

**DOI:** 10.34133/bmef.0110

**Published:** 2025-03-13

**Authors:** Zhou Fang, Kelsey Krusen, Hannah Priest, Mingshuang Wang, Sungwoong Kim, Anirudh Sriram, Ashritha Yellanki, Ankur Singh, Edwin Horwitz, Ahmet F. Coskun

**Affiliations:** ^1^ Wallace H. Coulter Department of Biomedical Engineering, Georgia Institute of Technology and Emory University, Atlanta, GA, USA.; ^2^ Machine Learning Graduate Program, Georgia Institute of Technology, Atlanta, GA, USA.; ^3^ Woodruff School of Mechanical Engineering, Georgia Institute of Technology, Atlanta, GA, USA.; ^4^ Interdisciplinary Bioengineering Graduate Program, Georgia Institute of Technology, Atlanta, GA, USA.; ^5^ Parker H. Petit Institute for Bioengineering and Bioscience, Georgia Institute of Technology, Atlanta, GA 30332, USA.; ^6^ Department of Pediatrics, Emory University School of Medicine, Atlanta, GA 30322, USA.

## Abstract

**Objective:** We developed 3-dimensional spatially resolved gene neighborhood network embedding (3D-spaGNN-E) to find subcellular gene proximity relationships and identify key subcellular motifs in cell–cell communication (CCC). **Impact Statement:** The pipeline combines 3D imaging-based spatial transcriptomics and graph-based deep learning to identify subcellular motifs. **Introduction:** Advancements in imaging and experimental technology allow the study of 3D spatially resolved transcriptomics and capture better spatial context than approximating the samples as 2D. However, the third spatial dimension increases the data complexity and requires new analyses. **Methods:** 3D-spaGNN-E detects single transcripts in 3D cell culture samples and identifies subcellular gene proximity relationships. Then, a graph autoencoder projects the gene proximity relationships into a latent space. We then applied explainability analysis to identify subcellular CCC motifs. **Results:** We first applied the pipeline to mesenchymal stem cells (MSCs) cultured in hydrogel. After clustering the cells based on the RNA count, we identified cells belonging to the same cluster as homotypic and those belonging to different clusters as heterotypic. We identified changes in local gene proximity near the border between homotypic and heterotypic cells. When applying the pipeline to the MSC–peripheral blood mononuclear cell (PBMC) coculture system, we identified CD4^+^ and CD8^+^ T cells. Local gene proximity and autoencoder embedding changes can distinguish strong and weak suppression of different immune cells. Lastly, we compared astrocyte–neuron CCC in mouse hypothalamus and cortex by analyzing 3D multiplexed-error-robust fluorescence in situ hybridization (MERFISH) data and identified regional gene proximity differences. **Conclusion:** 3D-spaGNN-E distinguished distinct CCCs in cell culture and tissue by examining subcellular motifs.

## Introduction

Spatial omics technique allows subcellular localization of important biomolecules. Such methods capture the spatial resolution lost during bulk and single-cell omics analyses due to sample processing. RNA fluorescence in situ hybridization (RNA-FISH) methods enable the identification of single RNA transcripts in human cells and tissue samples at single-transcript resolution [1]. Sequential FISH (seqFISH) and multiplexed-error-robust FISH (MERFISH) empower multiplexed detection of transcripts in the same sample and can label between 100 and 10,000 genes [2–4]. Common analysis methods approximate cells as a 2-dimensional (2D) projection on the cell growth surface or tissue sample adherent surface. However, ignoring 3-dimensional (3D) spatial resolution can alter the relative spatial relationship between transcripts (Fig. S1). Therefore, analysis using 3D spatial information provides the highest spatial accuracy. 3D imaging becomes possible with advancements in experiments and imaging techniques [5,6]. Therefore, further analyses are required to extract information encompassed by the data.

Graphs can represent various forms of data in biological contexts, such as protein signaling networks and gene regulation networks [7]. Multiple analysis methods that utilize graph representation of spatial omics data have been developed. For example, spaGCN represents spatial transcriptomics data in a graph based on spatial distance, and uses a graph convolutional network to integrate spatial transcriptomics and histology information to discover spatial domains [8]. GraphST represents spatial transcriptomics data in nearest neighbor graphs and applies self-supervised graph neural networks (GNNs) to embed the data into relevant latent features [9]. However, these methods only utilize local gene expression levels in spatial transcriptomic data. The subcellular distribution of RNA transcripts was ignored.

Organoids, spheroids, and aggregates have utilized various synthetic extracellular matrix (ECM)-like 3D scaffold to support stem cell functions [10]. A recent formulation of four-armed poly(ethylene glycol) macromer with maleimide groups at each terminus (PEG-4MAL) hydrogel exhibited better physiological relevance compared to mouse-based Matrigel platforms for 3D cell cultures [11–14]. Bioadhesive hydrogels incorporate unique peptides to mimic specific ECM proteins [15–17]. For example, GFOGER peptide mimics collagen I as a 3D network distributed across the cell cultures up to 0.5 mm above the glass, providing efficient stem cell growth and differentiation [17]. ECM-like gel can also be extracted from natural sources. For example, Cultrex gel derived from Engelbreth–Holm–Swarm, which contains a mixture of collagen and laminin, has been used to provide ECM support for cell culture and construct 3D cell models [18,19]. Coculture system studies can also benefit from 3D spatial transcriptomics. Coculture studies allow systematic measurement of cell–cell communication (CCC) [20,21]. However, the complex 3D system can lose complexity if approximated as 2D. Thus, rigorous analysis in 3D space is needed.

Paracrine cell signaling can happen through cell–cell direct contact [22,23] and secretion of signaling molecules [24,25]. Paracrine signaling regulates the cellular function and fate decisions to assist the multicellular organization [26]. High-throughput molecular analyses contain data about CCCs. Several computational methods have been developed to model paracrine signaling and infer potential CCCs using single-cell RNA sequencing and spatial transcriptomics data [27,28]. Spatially resolved multi-omics have shown promise in studying and visualizing subcellular and intercellular pathways and studying the activation of each signaling pathway [28,29] (Table). However, these methods utilize the limited spatial context of cells. They too lack the information from subcellular spatial distribution of genes. The subcellular distribution of genes has been shown to correlate with many cell functions, such as cell cycle regulation [3,30] and protein translational regulation [31].

**Table.** Comparison of existing CCC inference methods

Method	General idea	Key assumptions
cellChat V2 [79]	Mass action based on scRNAseq and existing ligand–receptor pairs, spatial distance to limit expected range of communications	Correlation of ligand–receptor expression indicates cell–cell interaction. Cell–cell interaction only happens at short distance
iTALK [80]	Correlation of ligand–receptor pairs	High coexpression of ligand–receptor between 2 cells indicates signaling
SoptSC [81]	Cell pairs scored by ligand–receptor correlation, but cells that show higher downstream gene expression are given more weight	Cell–cell communication happens through ligand–receptors, but cells that receive the signal need to react to the signal to form the signal chain
stLearn [82]	Local hotspots of ligand–receptor pair correlation	
CytoTalk [83]	Construct intracellular networks, then connect networks with ligand–receptor defined cross-talk edges	Intracellular signal transduction pathway needs to be elevated by both sender and receiver to be considered signaling
DeepTalk [84]	Construct intercellular/interspot graphs using ligand–receptor interaction in close range, then train graph attention network to reconstruct the subgraph. The trained model was then used to predict CCC.	Spatially proximal coexpression of ligand–receptor pairs indicates possible CCCs. Elevated expression of downstream genes and transcription factors indicates strong CCC effects.
SpaTalk [85]	Decompose the spatial transcriptomics spot using scRNAseq. Then, scoring neighboring ligand–receptor interactions based on coexpression in neighboring cells. Then, identify intracellular effect using random-walk in the knowledge-graph involving ligand–receptor and transcription factors-target.	Spatially proximal coexpression of ligand–receptor pairs indicates possible CCCs. Elevated expression of downstream genes and transcription factors indicates strong CCC effects.

**Fig. 1.**
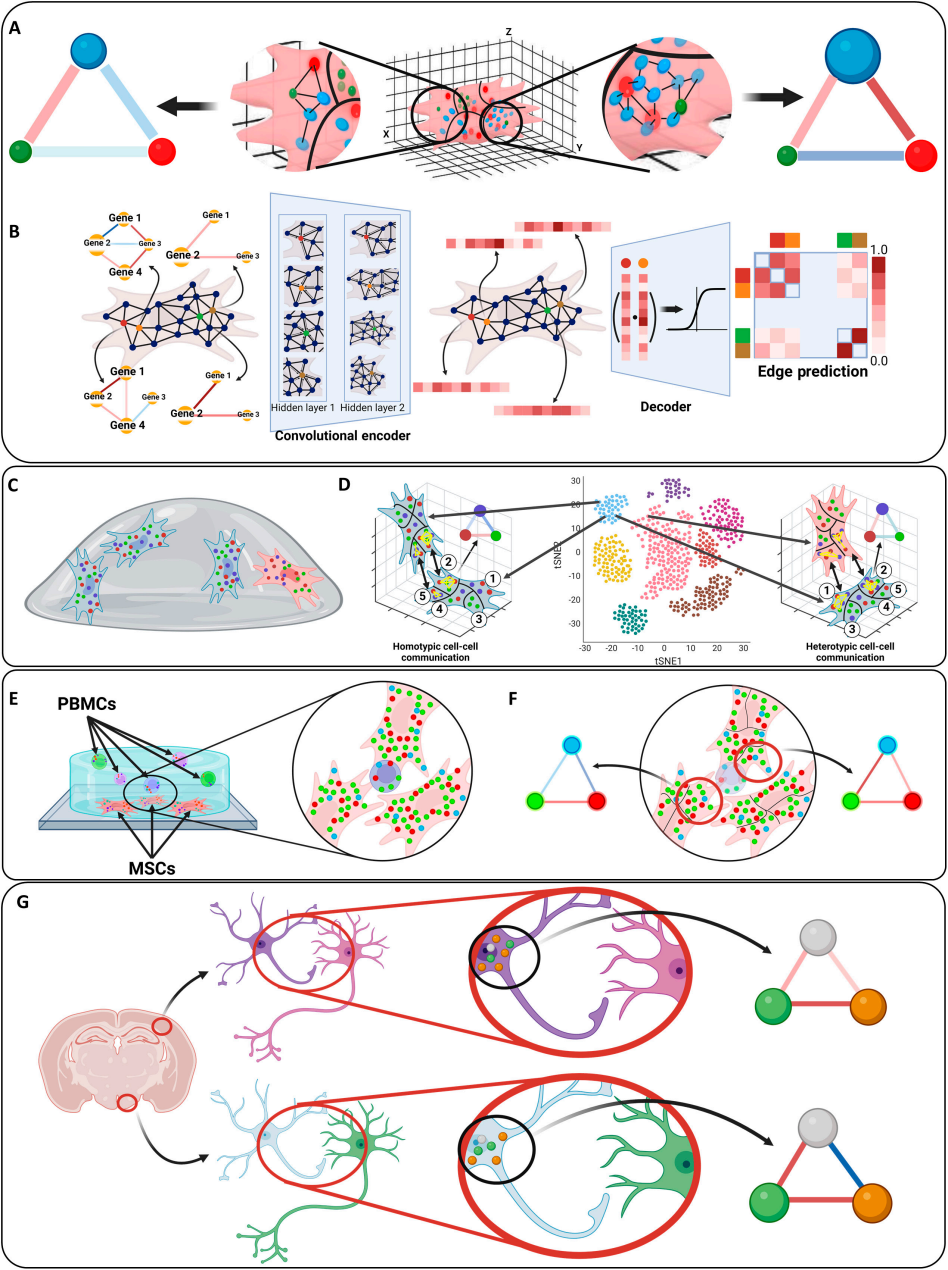
3D-spaGNN-E for examining CCC in MSCs cultured in gel, MSCs cocultured with immune cells, and astrocyte–neuron CCC. (A) 3D-spaGNN-E utilizes 3D localization of transcripts to compartmentalize cells into subcellular patches and computes the gene proximity score of each subcellular patch. Created in BioRender (Coskun A, 2025; https://BioRender.com/q17a642). (B) Graph autoencoder was trained to reconstruct the original graph and embed the gene proximity of subcellular patches into latent space. Created in BioRender (Coskun A, 2025; https://BioRender.com/a84i753). (C) 3D spatially resolved subcellular transcriptomics at single-transcript resolution. Created in BioRender (Fang Z, 2025; https://BioRender.com/v08v203). (D) Differential CCC between homotypic and heterotypic MSCs in the gel. The neighboring cell pairs are labeled as homotypic or heterotypic based on the clustering results using single-cell RNA count. We then compared the gene proximities and latent space features of the border patches. Created in BioRender (Fang Z, 2025; https://BioRender.com/e90b054). (E) Coculture of MSCs and PBMCs in gel. The coculture allows MSCs and PBMCs to interact under in vitro conditions. Created in BioRender (Coskun A, 2025; https://BioRender.com/n08j765). (F) CCC between MSCs and PBMCs in gel. We examined the gene proximity and latent space embedding of the MSC patches that border the PBMCs. Created in BioRender (Fang Z, 2025; https://BioRender.com/w22s855). (G) Study of CCC in 3D tissue. We analyzed MERFISH data to study differences between astrocyte–neuron CCC between the hypothalamus and cortex. Created in BioRender (Fang Z, 2025; https://BioRender.com/o42h892).

To address the above issue, we developed 3D spatially resolved subcellular gene neighborhood network (spaGNN) embedding, termed 3D-spaGNN-E. We further advanced the spaGNN [32]. The RNA transcripts were first detected in 3D location. Each cell is separated into spatially resolved patches using clustering. Then, we quantified the pairwise gene proximity relationships within each patch using permutation analysis (Fig. 1A). To further extract features from the spatial distribution of gene proximity relationships, we constructed a nearest neighbor graph between the center positions of each spatially resolved patch and applied a graph autoencoder to embed the proximity relationships into a latent space (Fig. 1B). We then analyzed the gene proximity changes and embedding changes of patches that face another cell to identify potential sites of CCC. We applied the 3D-spaGNN-E pipeline to MSC cultured in 3D hydrogel (Fig. 1C) and analyzed CCC between similar and different subtypes of MSCs (Fig. 1D). We then established MSC-PBMC coculture models in gel (Fig. 1E) and studied CCC between MSCs and CD4^+^ and CD8^+^ T cells (Fig. 1F). Lastly, we applied the pipeline to published 3D MERFISH data in mouse brain, and compared astrocyte–neuron CCC in the hypothalamus and the cortex (Fig. 1G).

## Results

### Multiplexed seqFISH-HCR obtains 31-plex spatially resolved subcellular transcriptional profile of MSCs in hydrogel

**Fig. 2.**
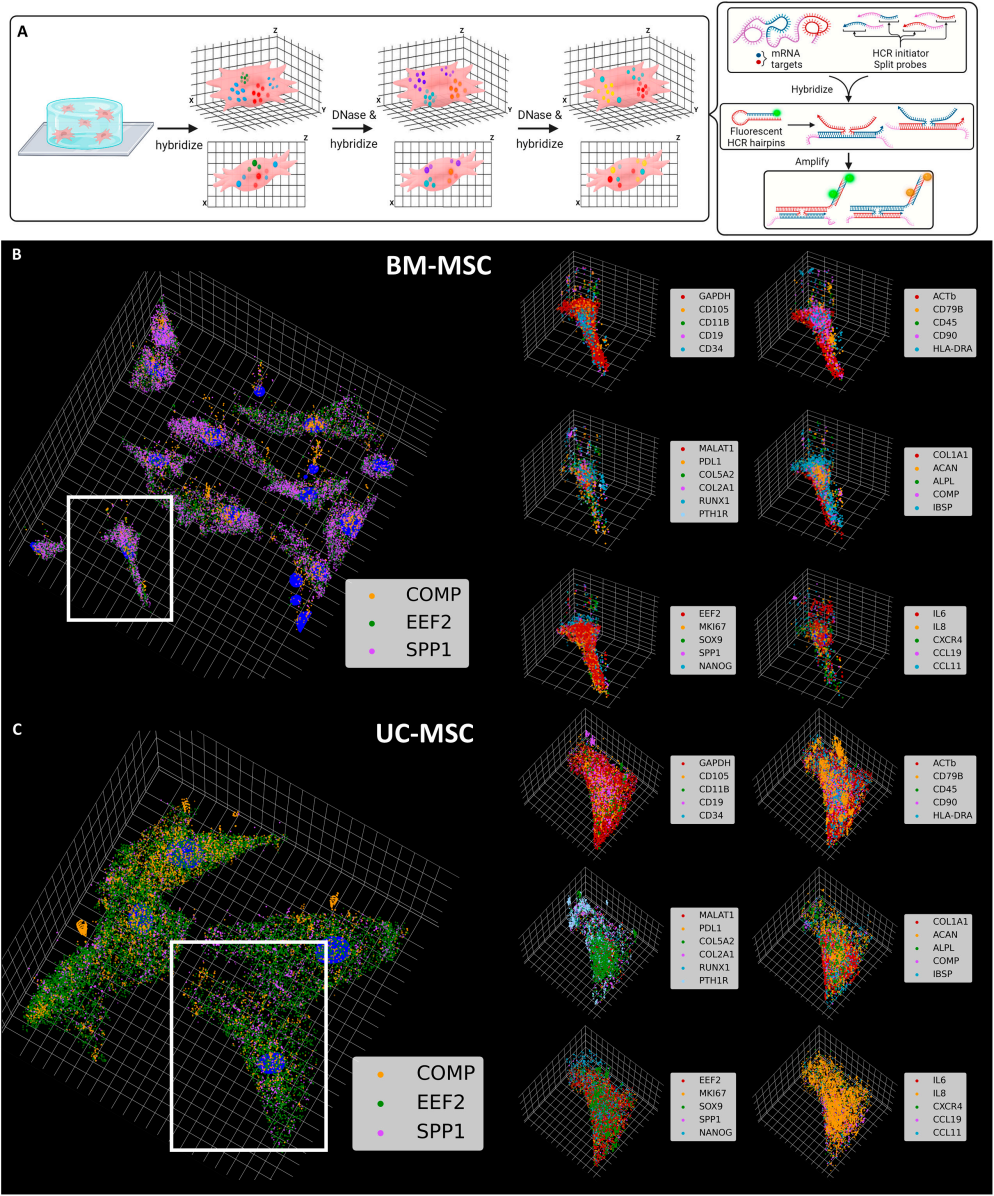
Sequential HCR-FISH in MSCs in hydrogel. (A) Sequential HCR-FISH of MSCs in 3D hydrogel. MSCs were embedded in hydrogel. Single transcripts were labeled using HCR-FISH. Amplification of fluorescence from HCR-FISH enhances the signal. The hybridization–DNase–hybridization cycle allows multiplexed detection of single transcripts. Created in BioRender (Coskun A, 2025; https://BioRender.com/h19e829). (B) 3D subcellular spatial transcriptomics profile of BM-MSCs. The original image contains diffraction-limited fluorescent dots. The dots were then detected, and the 3D positions were plotted in a scatterplot. The color legend indicates the color of each gene. A detailed view of the highlighted cell with 6 genes per plot is shown in the panel on the right. (C) 3D subcellular spatial transcriptomics profile of UC-MSCs. The subfigure is shown in the safe format as (B). The grid in all 3D scatterplots is 10 μm per line in both *x* and *y* axes and 1 μm per line in *z* axis.

Multiplexed RNA-FISH was performed in 3D hydrogels with high stiffness (10% w/v, PEG-MAL) using the sequential labeling, imaging, and re-hybridization strategy. A library of 31 RNA targets 5-d cultured and fixed bone marrow-derived mesenchymal stem cells (BM-MSCs) and umbilical cord-derived MSCs (UC-MSCs) located across the 30-μm-thick hydrogel layer using up to 100 optical slices on a wide-field fluorescence microscope (Fig. 2A). We utilized the third-generation hybridization chain reaction (HCR) to increase the fluorescence strength [33]. The stronger fluorescence penetrates through the gel, allowing imaging of cells deeper in the gel. The image stacks capture the same diffraction-limited dots at varying intensity across several slices. We first locate the diffraction-limited dots in a maximum-intensity projected image across the *z* axis. By identifying the slice with the highest intensity at the 2D position of each transcript, we located the depth of the detected transcript. Such imaging and detection processes provide 3D volumetric distributions of multiple RNA species in a single measurement. The positions of the detected transcripts were then plotted along with the cell nuclei (Fig. 2B and C and Figs. S2 and S3). We captured a 31-plex spatially resolved transcriptomics profile at single-transcript resolution while maintaining the spatial context of each cell and its RNA transcripts.

### 3D-spaGNN-E analysis detects subcellular gene proximity relationship

**Fig. 3.**
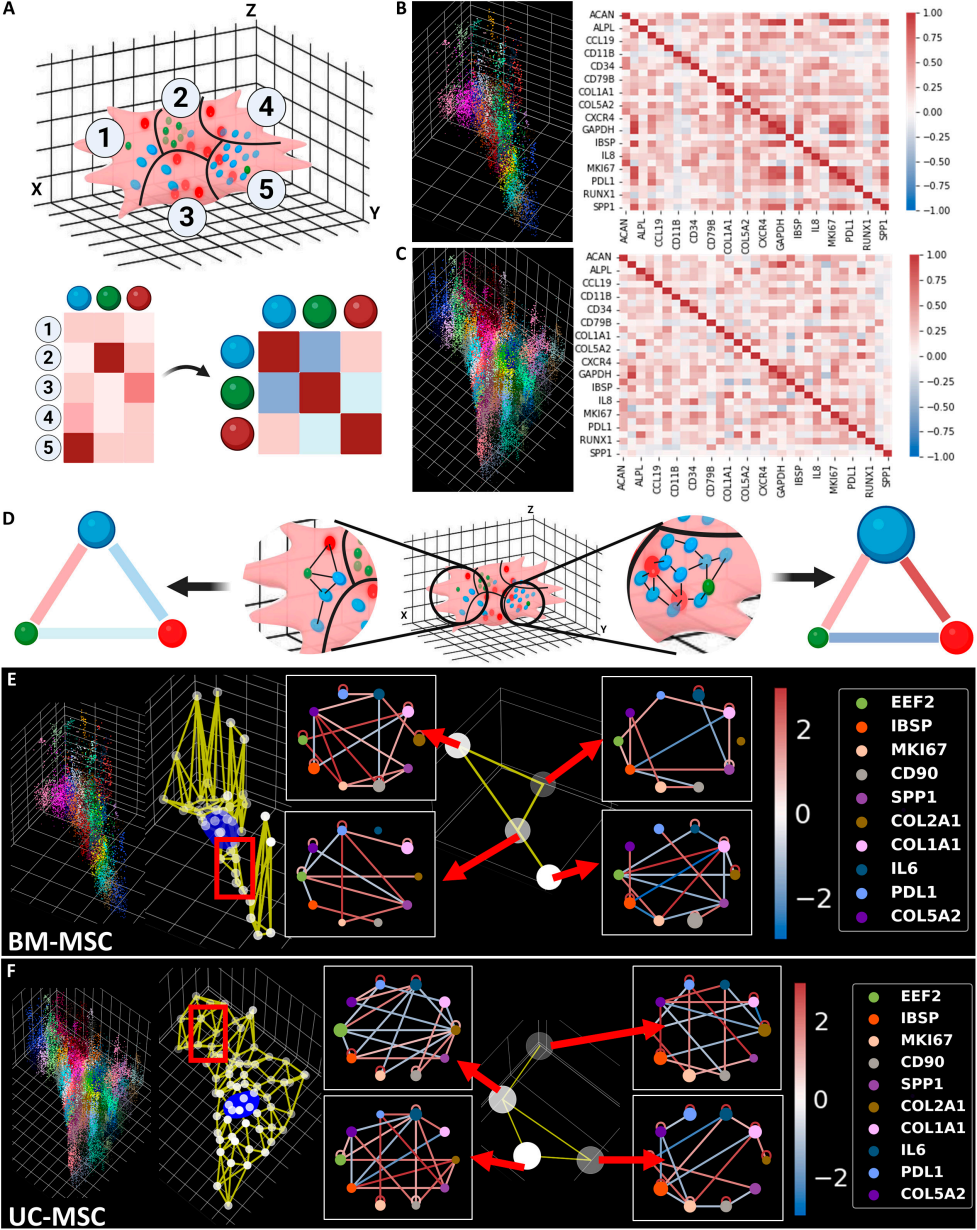
SpaGNN analysis of MSCs in 3D. (A) Patch correlation of MSCs in 3D. Cells were separated into spatially resolved patches by clustering the 3D positions of all detected transcripts. The copy number of each gene in each patch was calculated (bottom left), and the correlation between genes among patches was computed. The pairwise gene correlation is shown in a heatmap (bottom right). Created in BioRender (Fang Z, 2025; https://BioRender.com/c98a775). (B) Example of BM-MSC patch correlation. The colored scatterplot shows the patch separation of a BM-MSC. Each dot indicates a detected transcript, and each unique color indicates an identified patch. Each grid in *x* and *y* axes is 10 μm, and each grid in *z* axis is 2 μm. The gene count of each patch was calculated. The pairwise Pearson’s correlation was calculated and shown in the heatmap on the right, where the color corresponds to the color bar. (C) Example of UC-MSC patch correlation. Each grid in *x* and *y* axes is 10 μm, and each grid in *z* axis is 2 μm. The analysis process to generate this panel is the same as the previous panel. (D) Pairwise gene proximity relationship within a patch. By finding the nearest neighbor graph and permutation analysis, we compute the pairwise gene proximity score that quantifies the tendency of transcripts to be immediate neighbors of each other. Created in BioRender (Coskun A, 2025; https://BioRender.com/q17a642). (E) Example of heterogeneous gene proximity of a BM-MSC. The patch separation is shown in the scatterplot on the left. The center position of each patch is shown in a white scatterplot. The patch and its nearest 2 neighbors were connected to form the patch graph. The patches in the highlighted region of the nearest neighbor graph and the gene neighborhood network involving selected genes are shown on the right. An edge is only drawn between genes with the absolute value of proximity score greater than 0.5. (F) Example of heterogeneous gene proximity of a UC-MSC. The grid in all 3D scatterplots is 10 μm per line in both *x* and *y* axes and 1 μm per line in *z* axis.

Our previous pipeline, spaGNN, extracts subcellular gene colocalization and pairwise proximity relationships. We expanded the existing spaGNN pipeline into 3D-spaGNN-E by incorporating the 3D position of the detected transcripts. 3D-spaGNN-E separates cells into spatially distinct patches by applying clustering on the positions of all detected RNA transcripts in the same cell. Each patch can contain transcripts of multiple genes. Therefore, we counted the copy number of each gene within each patch and computed the pairwise correlation between genes among all patches in the same cell (Fig. 3A). Examples of this analysis performed in BM-MSCs and UC-MSCs are shown in Fig. 3B and C. The colored scatterplot shows the patch separation of single cells, with each color corresponding to a spatially unique subcellular patch (Fig. S4). The heatmap shows the calculated Pearson’s correlation between each pair of genes. The correlation value ranges between −1.0 and 1.0, and corresponds with the blue–red color bar shown on the right.

Within each patch, we then established local gene proximity relationships by constructing a nearest neighbor graph among transcripts in each patch. We utilized a permutation analysis to compute the pairwise proximity score z. The permutation analysis shuffles the gene of each transcript 500 times. The number of connections in the nearest neighbor graph between 2 genes was counted for each permutation. The permutation process finds the mean and standard deviation of the number of neighboring events between two genes if the transcripts’ spatial distribution were random. The *z* score of the observed number of connections between 2 genes was computed as the proximity score. Thus, the proximity score indicates a pair of genes to be more or less likely to be neighbors than random chance. We then visualized the analysis result in network form (Fig. 3D). Examples of heterogeneous subcellular gene proximity from BM-MSCs and UC-MSCs are shown in Fig. 3E and F (Figs. S5 and S6). The colored patch separation of single cells is shown in the left panel. We then extracted the center positions of each patch and connected each patch with its 2 nearest neighbors to form a patch nearest neighbor graph for future analysis. Several patches and their gene neighborhood networks are shown in Fig. 3E and F. The node of the network is colored by gene according to the color legend, and the sizes of the node indicate the enrichment of the gene in the patch. An edge is connected between a pair of genes if $|z_{\text{gene}1,\text{gene}2}|>0.5$, meaning we consider gene neighbor counts between gene pairs within one SD of the expected number of gene neighbors to be no substantial differences from random.

### Graph autoencoder embeds subcellular proximity features into a latent space

We applied a graph autoencoder to extract higher-level features describing the gene proximity relationships in a cell [34]. The graph autoencoder encodes the original features of each node into a latent space and decodes the latent space features with a decoder to reconstruct the original graph. Such methods have been used to process and analyze spatially resolved transcriptomics and proteomics data [35–37]. We seek to apply the graph autoencoder to subcellular spatial transcriptomics.

**Fig. 4.**
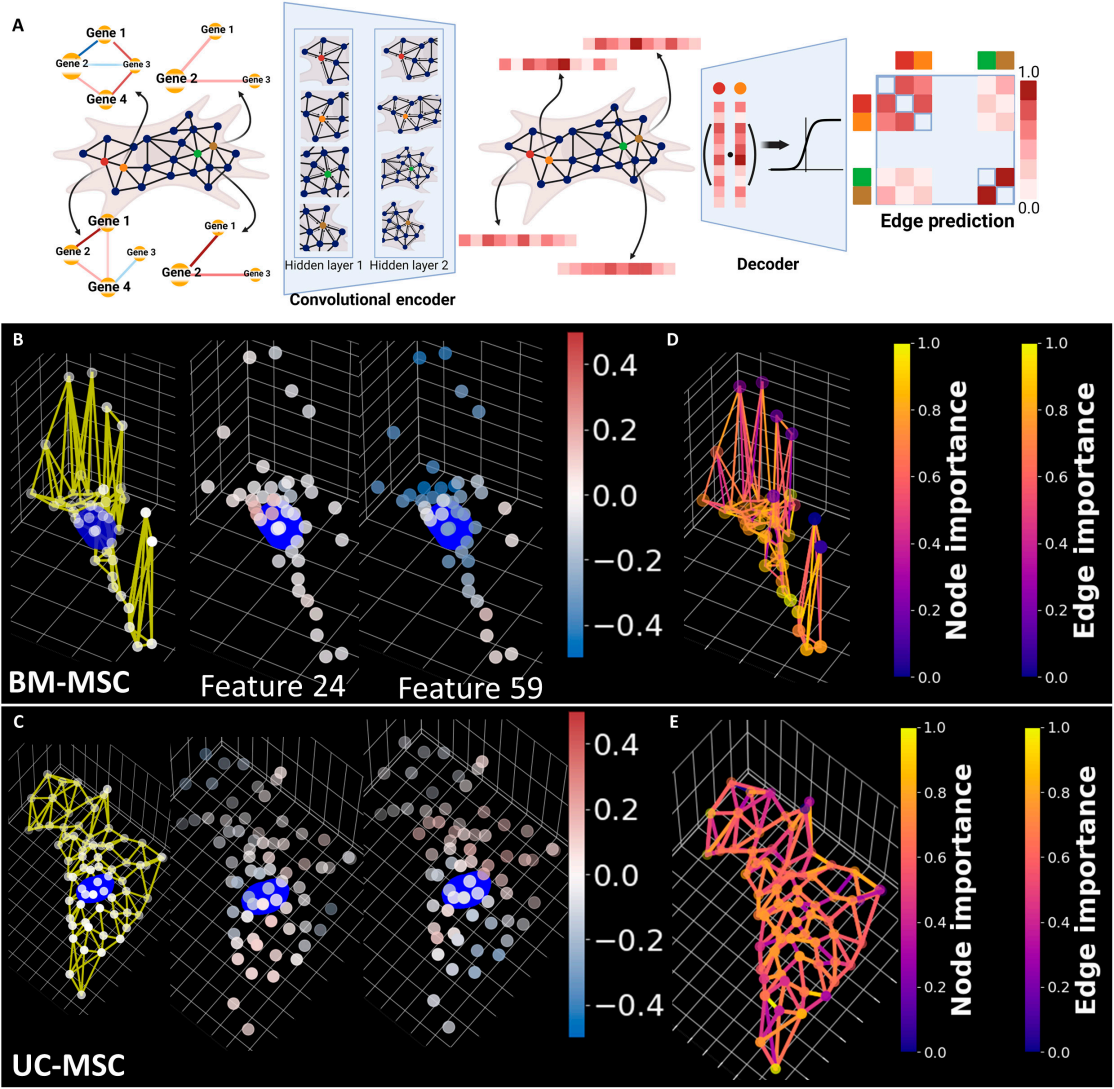
Graph autoencoder for embedding gene proximity into latent space. (A) Graph autoencoder to embed the patch graphs constructed from single cells. Patch graphs were constructed by defining each patch as nodes, and connecting each patch and its 3 nearest neighbors as edges. The pairwise gene proximity scores of each patch visualized by the gene neighborhood network are defined as the node features. The convolution layers encode the node features into a new set of features of lower dimension. The inner product decoder uses the encoded features to compute the probability of an edge between every pair of nodes. The edge predictions were then compared to the actual edges, and the parameters in the encoder were adjusted. In the illustration, the trained autoencoder predicted a high probability of brown–green and red–orange connections, and a low probability of brow–red, brown–orange, green–red, and green–orange connections. The predicted results match the original graph. Created in BioRender (Coskun A, 2025; https://BioRender.com/a84i753). (B) Embedded features of patches of a BM-MSC. The features were selected based on the significant differences between BM-MSCs and UC-MSCs. The embedded feature values of each node correspond to the color of the node. (C) Embedded features of patches of a UC-MSC. The feature values correspond to the color of the node. (D) The node importance of the BM-MSC is shown in (B). A GNN explainer model was used to find important nodes and edges that influence the embedded features. The node and edge importance correspond to the color bar. Nodes and edges with importance values close to one have a high influence on the embedded features. (E) The node and edge importance of the UC-MSC is shown in (C). The grid in all 3D scatterplots is 10 μm per line in both *x* and *y* axes and 1 μm per line in *z* axis.

The input graph for the autoencoder requires nodes and edges that connect selected pairs of nodes. Features of each node are required. Therefore, we use the center positions of patches as node positions and the pairwise gene proximity relationships as node features. The nodes were connected to their 2 nearest neighbors to form a patch nearest neighbor graph. We then constructed a 2-layer convolutional encoder and an inner product decoder (Fig. 4A). The encoder projects each node’s pairwise gene proximity score into a latent space while also reducing the dimensions. The numbers of output channels of both the convolutional layers were tuned using a grid search (Fig. S7). The decoder applies a sigmoid function to the inner product of the latent features of any pair of nodes to predict an edge between the pair of nodes, and the error between the predicted edges and the true edges was backpropagated to train the convolutional layers.

Examples of the encoded patch nearest neighbor graph of single cells are shown in Fig. 3B and C. The original proximity scores between 480 unique gene pairs in each node were projected to 100 features in a latent space. The embedded values of several features of nodes in the same cell are shown in the middle panes in Fig. 4B and C. The 2 features shown are among features with significant differences between BM-MSCs and UC-MSCs (Fig. S8). We also performed cell clustering and classification based on the pooled embedding values. We pooled embedded features of all nodes within the same cell by computing the average of each feature. We then compared the clustering and classification of cells as BM-MSCs or UC-MSCs using single-cell RNA count, patch gene correlation, variation in local gene neighborhood networks, and pooled embedded features (Figs. S9 and S10). We used logistic regression, support vector machine (SVM), random forest, and *k*-nearest neighbor (kNN) classifier to compare the classification of cells. All classifiers can separate the cells based on the previously mentioned features. Both logistic regression and SVM show similar performances using all 4 sets of features. However, random forest shows the lowest accuracy and area under the receiving operating curve (AUC) using the autoencoder embedded features, and kNN shows the lowest accuracy and AUC using the variation of gene proximity networks.

The classification analysis shows that the encoded features present separation between BM-MSCs and UC-MSCs. The convolutional layer utilizes message passing, thus allowing the embedded feature to incorporate features of surrounding nodes. Differences between BM-MSCs and UC-MSCs in embedded features indicate the presence of local similar patch nearest neighbor graphs. Therefore, we applied a GNN explainer to find edges, pairwise proximity scores, and nodes that change the embedded features [38] (Fig. 4D and E). The figures highlight the high-importance nodes and edges.

### Homotypic and heterotypic CCC between MSCs in hydrogel

Cells communicate with each other through various methods, including soluble factors and direct contact. CCC can impact cells’ fate decisions and phenotypes [39,40]. Previous studies have categorized CCCs as homotypic and heterotypic CCCs. Homotypic CCCs are communications between the same cell type, and heterotypic CCCs are communications between different cell types. Homotypic and heterotypic CCCs can have different effects on cell fate and proliferation. Previous studies have shown that both homotypic and heterotypic CCCs are critical for cell proliferation, angiogenesis, and cell differentiation [41–43]. Existing studies have also used coculture systems and microfluidic chips to model CCCs [44,45].

**Fig. 5.**
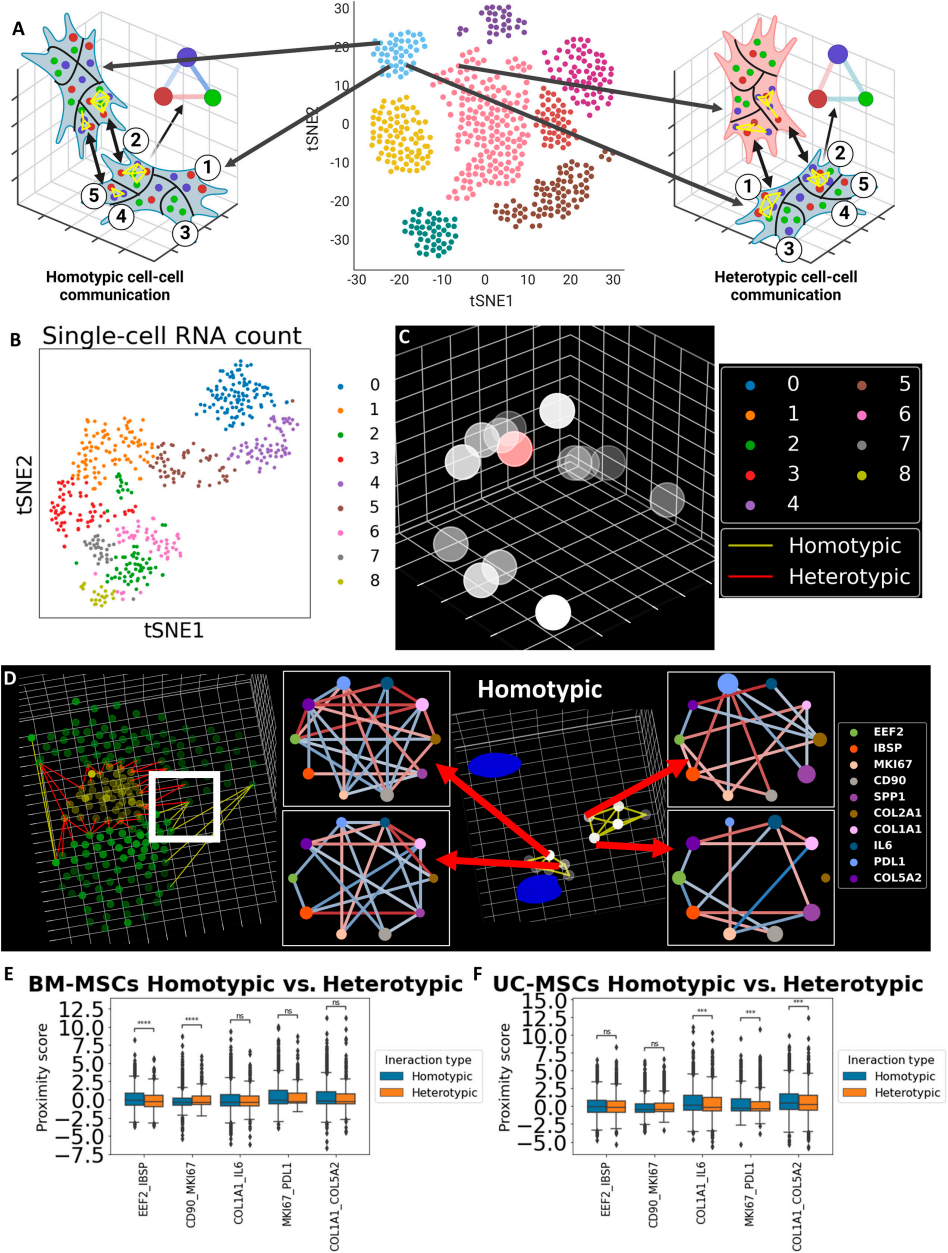
Homotypic and heterotypic cell interaction between MSCs in 3D hydrogel. (A) Homotypic and heterotypic interaction between MSCs in the 3D hydrogel. The cells were clustered based on single-cell RNA count. Immediate spatial proximity between cells was considered a cell–cell interaction. The interaction is considered homotypic if the 2 cells are of the same cluster, and heterotypic if the cells are of different clusters. Created in BioRender (Fang Z, 2025; https://BioRender.com/e90b054). (B) tSNE plot of the clustering of cells based on single-cell RNA count. The MSCs were clustered based on the single-cell RNA count. The clustering results are visualized in a tSNE plot where each scatter point represents a cell and the position of the point represents the tSNE embedding of the RNA count of the cell. (C) Location of captured CCC in 3D hydrogel. Images were captured at multiple positions of the hydrogel. The cells imaged at the highlighted region are shown in (D). (D) Examples of homotypic and heterotypic interactions. The scatter points show the center positions of detected patches of each cell. The scatter point colors correspond to the cluster of the cell. A Voronoi graph was generated among all subcellular patches within the same imaging field of view. Patches from different cells that are connected by an edge are considered border patches. Edges that connect patches of 2 cells from the same cluster are defined as homotypic interactions, and edges that connect patches of cells from different clusters are defined as heterotypic interactions. Yellow edges indicate identified homotypic interactions and red edges indicate identified heterotypic interactions. The *x* and *y* axes have grid spacing of 10 μm, and the *z* axis has grid spacing of 1 μm. (E) Boxplot comparing proximity scores of border patches between BM-MSCs. Homotypic border patches show higher EEF2–IBSP and lower COMP–MKI67 proximity. (F) Boxplot comparing proximity scores of border patches between UC-MSCs. Homotypic border patches show higher COL1A1-IL6, MKI67-PDL1, and COL1A1-COL5A2 proximity. The statistical significance annotation is as follows: *P* value annotation legend: ns: 5.00 × 10^−2^ < *P* ≤ 1.00, *: 1.00 × 10^−2^ < *P* ≤ 5.00 × 10^−2^, **: 1.00 × 10^−3^ < *P* ≤ 1.00 × 10^−2^, ***: 1.00 × 10^−4^ < *P* ≤ 1.00 × 10^−3^, ****: *P* ≤ 1.00 × 10^−4^.

Thus, we seek to find local gene proximity relationships that indicate homotypic and heterotypic CCCs between MSCs. We first performed clustering on 553 imaged MSCs based on the single-cell RNA count to identify homotypic and heterotypic cells (Fig. 5A). We then defined cells of the same cluster as homotypic and cells of different clusters as heterotypic. We detected 9 clusters, clusters 0 to 8, in the clustering analysis (Fig. 5B and Fig. S11A). Each cluster shows varying marker genes. For example, cluster 0 shows elevated expression of osteogenic markers such as SPP1 and IBSP; cluster 5 shows elevated cytokine expression such as interleukin-8 (IL-8); and cluster 8 shows elevated expression of ECM genes such as COL1A1 and COL5A2 (Fig. S11B).

Within the same field of view, we define cells with no other objects between them as communicating cells. We captured multiple fields of view across the same piece of hydrogel, thus identifying multiple potential CCC sites (Fig. 5C). We generated a Voronoi graph among all subcellular patches of all the cells in the same field of view. We used only *x* and *y* positions of each patch to construct the Voronoi graph, as constructing a Voronoi graph using the 3D positions connects nodes on top of each cell. These patches would be considered immediate neighboring by the Voronoi graph but are far apart. If a Voronoi ridge connects a pair of patches from different cells, the 2 cells are considered interacting. We define interacting cell pairs of the same cluster as homotypic and cell pairs of different clusters as heterotypic (Fig. 5D). We defined patches that connect with another cell as border patches. The patch that connects with a homotypic cell was defined as a homotypic patch, and the patch that connects with a heterotypic cell was defined as a heterotypic patch. We identified 9,611 patches as border patches among 25,441 subcellular patches. We then examined the gene proximity of both homotypic and heterotypic patches. The local regions around border patches and the gene neighborhood networks were shown. Further examples of homotypic and heterotypic patches between BM-MSCs and between UC-MSCs are shown in Figs. S2 to S15.

We performed statistical analysis to compare the gene proximity of the border patches. We performed a homotypic versus heterotypic comparison for both BM-MSCs and UC-MSCs. The comparison shows that homotypic and heterotypic patches of BM-MSCs and UC-MSCs differ in different gene pairs, with a 2-color heatmap visualizing gene pairs with significantly different proximity scores between heterotypic and homotypic CCCs (Fig. S16A and B). Boxplots of gene proximity scores of homotypic and heterotypic patches with statistical annotation are shown in Fig. 5E and F. The statistical annotation follows the legend: ns: 5.00 × 10^−2^ < *P* ≤ 1.00, *: 1.00 × 10^−2^ < *P* ≤ 5.00 × 10^−2^, **: 1.00 × 10^−3^ < *P* ≤ 1.00 × 10^−2^, ***: 1.00 × 10^−4^ < *P* ≤ 1.00 × 10^−3^, ****: *P* ≤ 1.00 × 10^−4^. In BM-MSCs, we observed gene pairs with significant differences in proximity scores between homotypic and heterotypic patches such as EEF2–IBSP and COMP–MKI67. Such differences were also observed for proximity scores of COL1A1–IL-6, MKI67–PDL1, and COL1A1–COL5A2 in UC-MSCs.

To visually validate the finding, we examined the positions of the original transcripts. Scatterplots of detected transcripts in 2 homotypic patches (Fig. S16C) and 2 heterotypic patches (Fig. S16D) of UC-MSCs, with COL1A1 and IL-6 highlighted, are shown. The proximity score was computed based on the nearest neighbor graph between transcripts within the same patch. Therefore, we also highlighted the edges of the nearest neighbor graph that connects COL1A1 transcripts and IL-6 transcripts. The scatterplot shows that homotypic patches present more COL1A1–IL-6 located in proximity, which aligns with the statistics discovery.

### Local CCC patch motif using GNN explainer

**Fig. 6.**
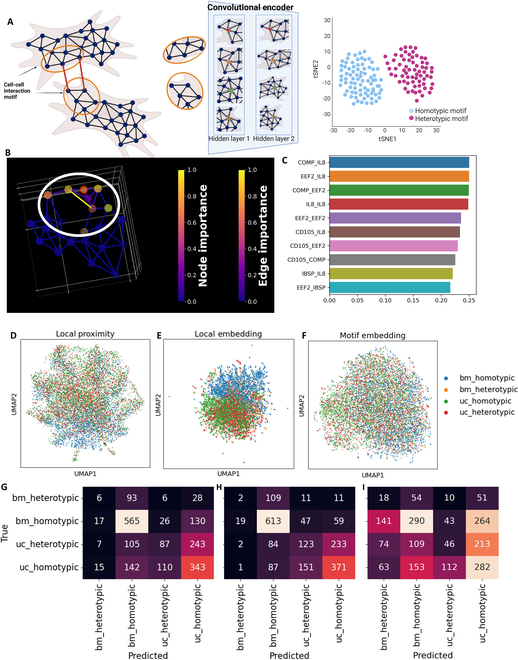
Embedding of CCC motif as classification of homotypic or heterotypic interaction. (A) Embedding of local cell interaction motifs using the graph autoencoder. The portion of the patch nearest neighbor graph around the border patches is defined as the cell interaction motifs. This analysis embeds the local cell interaction motifs with the autoencoder, and the embedded features are used to classify the border patch as homotypic or heterotypic. Created in BioRender (Fang Z, 2025; https://BioRender.com/t58n733). (B) GNN explainer algorithm detects the cell interaction motifs. A GNN explainer algorithm was applied to the encoder to find important gene proximity, patch nearest neighbor graph nodes, and edges. The patch and edges of high importance to embedding the border patch are defined as the cell interaction motif. The *x* and *y* axes have grid spacing of 10 μm, and the *z* axis has grid spacing of 1 μm. (C) High-importance gene proximities of cell interaction motifs. High importance indicates that the changes in the proximity cause higher changes in the embedding values. (D) UMAP of average pooled embedding of cell interaction motifs. (E) Uniform manifold approximation and projection (UMAP) of local pairwise gene proximity of border patches. (F) Confusion matrix of classifying cell types and cell interaction types using cell interaction motif embedding. (G) Confusion matrix of classifying cell types and cell interaction types using border patch gene proximity. (H) Confusion matrix for border patch embedding, and (I) pooled motif embedding.

The explainability of machine learning algorithms has been widely studied. Explainability analysis breaks the deep learning “black box” and finds the features in the original data that contribute to the final prediction of the model [46]. Explainability has been especially important in the biomedical field as it can find connections between original data and the prediction, as well as provide transparent and tracible predictions [47,48]. Previous applications of explainable GNNs have been shown to identify local immune cell motifs in secondary lymphoid tissue [49]. Thus, we seek to identify local CCC motifs using the GNNExplainer algorithm [38]. We have observed the autoencoder embedded features to show statistically significant differences between homotypic and heterotypic patches (Figs. S7 and S18). We defined the CCC motifs around homotypic patches as homotypic motifs, and CCC motifs around heterotypic patches as heterotypic motifs. We seek to embed the CCC motifs using the autoencoder to classify homotypic and heterotypic motifs (Fig. 6A).

We applied the GNNExplainer algorithm to the encoder section of the autoencoder to identify the edges and nodes that change the embedding of the border patches. The nodes and edges with high importance are identified as CCC motifs (Fig. 6C). We also analyzed the gene proximities that change the embedding of the border patch. COMP–IL-8, EEF2–IL-8, and COMP–EEF2 proximity shows the highest influence on the embedding of the border patch (Fig. 6C). We then extracted the CCC motifs and fed the CCC motifs into the autoencoder. We compared the classification performance of CCC using gene proximity of the border patch, the autoencoder embedding of the border patch, and the embedding of the CCC motifs. We labeled each motif or border patch by the cell types and CCC types (Fig. 6D to F). We then applied an SVM classifier to classify the CCC motif as BM_homotypic, BM_heterotypic, UC_homotypic, and UC_heterotypic motif using the border patch gene proximity (Fig. 6G), border patch embedding (Fig. 6H), and pooled motif embedding (Fig. 6I), and visualized the classification results using confusion matrices. The confusion matrix shows that the classification of BM_homotypic, UC_heterotypic, and UC_homotypic CCC was the most accurate using the embedding of the border patch. The pooled motif embedding performed the best when classifying BM_heterotypic CCC. The border proximity classification only considers the gene proximity near the border. The border patch embedding convolves the gene proximity of the nearby patches into the border patch embedding features, thus increasing the influence of the gene proximity in the interior patches in the classification features. The pooled motif embedding further increases the influence of the interior patches. Thus, we theorize that the gene proximity changes near the border of CCC, and the influence of the CCC on gene proximity decreases while moving to the interior. The autoencoder embedding incorporates the CCC-related proximity changes in the interior patches into the embedding of the border patch features.

### Coculture of MSCs and PBMCs presents potential cell interaction sites

MSCs have been shown to suppress immune cells in autoimmune diseases as well as allogeneic immune cells during organ transplantation [50,51]. MSCs can also be modified or primed before injection for treatment [52,53]. For example, interferon-γ (IFN-γ) has been shown to increase the immunomodulatory potential of MSCs [20,54]. The suppression mechanism of MSCs on immune cells can be attributed to both secreted molecules and direct contact [55]. The suppression of immune cells reduces the proliferation of immune cells. Therefore, the proliferation of immune cells can be used to indicate CCCs. Previous studies have used biomaterials to enable the coculture of multiple cell types to study cell interactions [56,57]. Thus, we seek to coculture human peripheral blood mononuclear cells (PBMCs) with MSCs to study CCC between MSCs and immune cells.

**Fig. 7.**
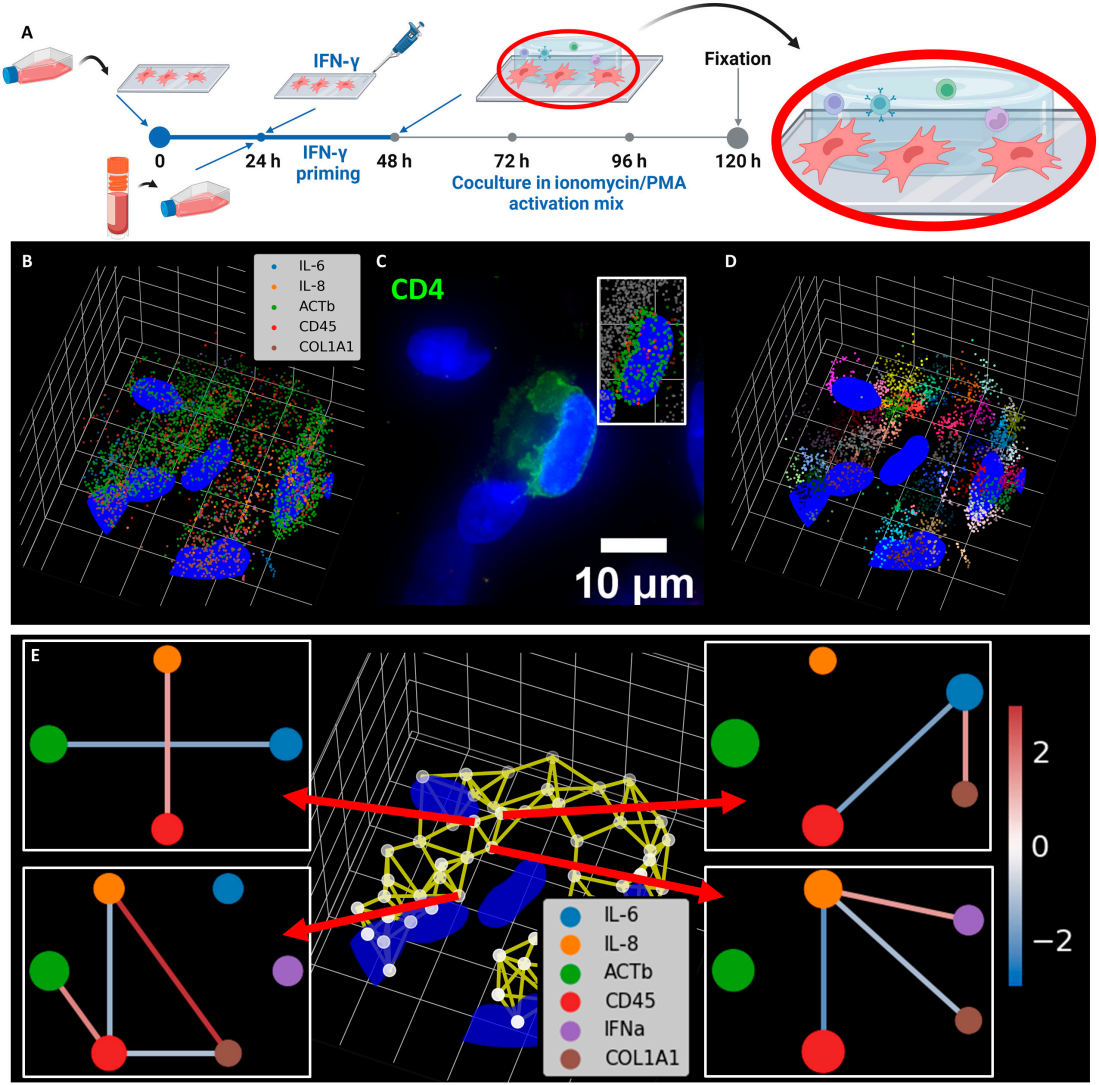
Applying coculture to investigate MSC–immune cell interactions. (A) Experimental scheme of MSC-PBMC coculture. BM-MSCs were seeded onto poly-l-lysine-treated coverslips. The MSCs were then primed with IFN-γ for 24 h. PBMCs were then embedded in Cultrex gel and seeded on top of the MSCs. The MSCs and PBMCs were cocultured for 72 h in an immune activation medium before fixation. Created in BioRender (Coskun A, 2025; https://BioRender.com/w74m863). (B) Multiplexed RNA-FISH data around gel-embedded immune cells. The blue color indicates the nuclei of the detected cells. Each scatter point represents a detected transcript, and the gene of the transcript is shown by the color legend. (C) Detected transcripts of a CD4^+^ T cell in 3D. An IF staining of CD4 detects the CD4^+^ T cells in the coculture. The transcripts of the detected T cell are shown in the highlighted box. (D) Spatial patches of the transcriptional microenvironment around the CD4^+^ T cell. All transcripts within 30 μm of an immune cell are considered the immune cell’s transcriptional microenvironment. We applied the Leiden clustering algorithm to the positions of transcripts to find spatially distinct patches of the transcriptional microenvironment. (E) Gene proximity of patches with the shortest distances to the immune cells. The patch nearest neighbor network of the transcriptional microenvironment is overlaid on the detected nuclei. We reason that the patch closest to the immune cell impacts the interaction between the MSCs and the immune cell. The gene proximity of patches that are closest to the immune cell is shown. All axes have grid spacing of 10 μm.

PBMCs were extracted from whole blood and cocultured with IFN-γ primed and unprimed BM-MSCs (Fig. 7A). The BM-MSCs were first cultured on poly-l-lysine-coated coverslips and primed with IFN-γ. After priming, PBMCs were mixed with Cultrex gel and seeded on the BM-MSCs. PBMCs and MSCs were cultured for 3 d before fixation and labeling. The multiplexed HCR-FISH protocol generated the subcellular-resolved RNA profile of the coculture system (Fig. 7B). To further understand the cell compositions of the coculture system, we performed immunofluorescence (IF) staining of immune markers (Fig. 7C). After identifying the immune cells, we analyzed the transcriptomic environment around each PBMC. We define all transcripts within 30 μm of the center PBMC as the transcriptional microenvironment. We then applied the 3D-spaGNN-E pipeline to find local gene proximity relationships (Fig. 7D). We then analyzed the local gene proximity of the patches closest to the immune cells (Fig. 7E).

To expand the data within a shorter time, we conducted 4 sets of multiplexed experiments with 6 genes labeled in each set of the experiment. The gene labeled in each set of the experiments is shown in (Table S1). Each set of experiments was analyzed separately. The IF staining identified 844 CD4^+^ T cells and 636 CD8^+^ T cells. Previous discussions have identified immune cell suppression as an indicator of CCCs. Therefore, we labeled the proliferation marker KI67 at the end of multiplexed HCR-FISH. High KI67 indicates high immune cell proliferation, low suppression, and weak CCC between MSCs and the immune cell. Examples of both KI67^+^ and KI67^−^ T cells from all 4 sets of experiments are shown in Figs. S19 and S20.

### Local gene proximity and autoencoder embedding of patch networks detect changes in gene localization at possible CCC sites between MSCs and T cell subsets

After analyzing the local gene proximity of proximal patches to the immune cells, we hypothesized that the MSC–immune CCC can differ between CD4^+^ and CD8^+^ T cells, and between KI67^+^ and KI67^−^ T cells. Therefore, we compared the gene proximity of 5 patches in the microenvironments nearest to the immune cell. The comparison shows that the proximal patches to CD4^+^ T cells have significantly lower COL1A1-HLA-DRA proximity and higher COL5A2-COL2A1 proximity (Fig. S21). Additional comparison shows CD4^+^ and CD8^+^ T cells of different KI67 expression levels. The results show that among CD4^+^ T cells, proximal patches to higher KI67-expressing cells show higher COL1A1–PDL1, HLA-DRA–PDL1, and PD1–GAPDH proximities, and lower IL-10–PD1, TNFα–COL2A1, and COL2A1–IFN-γ proximities. Among CD8^+^ T cells, the proximal patches to higher KI67-expressing cells show lower COL5A2–CXCL10 proximity (Fig. S22). Notably, the statistical comparison of patch proximity between KI67^+^ and KI67^−^ cells can be affected by the low number of KI67^+^ CD8^+^ T cells, causing more comparisons to be insignificant.

**Fig. 8.**
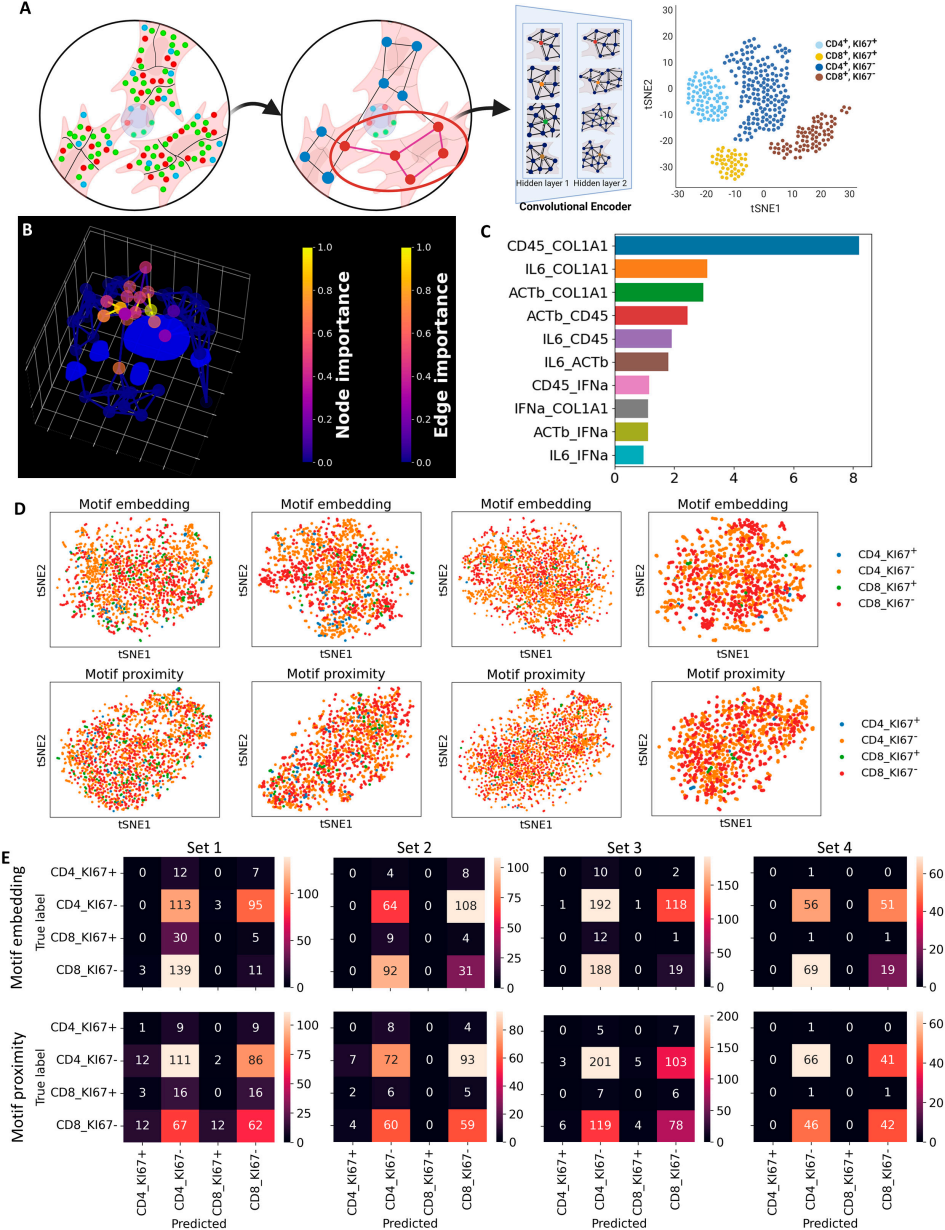
Embedding of CCC motif for classification of immune cell type and proliferation. (A) Identification and embedding of MSC–immune CCC motifs. The CCC patch motifs around the immune nearest patches were identified. The graph autoencoder projected the identified CCC motifs into the latent space. The embedded features were then pooled and clustered for classification. Created in BioRender (Fang Z, 2025; https://BioRender.com/s28d254). (B) Identification of MSC–immune CCC motifs using GNN explainer algorithm. The highlighted patches and edges indicate a CCC motif. All axes have grid spacing of 10 μm. (C) High-importance gene proximities. The GNN explainer also identifies the high-importance gene proximities that change the embedding of the CCC motifs. The gene proximity with the highest importance is CD45–COL1A1, meaning changes in CD45–COL1A1 proximity change the embedding the most. (D) tSNE plot of CCC motifs by pooled gene proximity (top row) and motif embedding (bottom row). The 4 columns of plots correspond to 4 sets of experiments with different gene studies in each set of experiments. The scatterplot shows the grouping of CCC motifs between MSC and CD4^+^ T cells and CD8^+^ T cells. However, the scatterplot shows low number of KI67^+^ T cells. (E) Confusion matrix of classification of CCC motifs by pooled gene proximity (top row) and motif embedding (bottom row). Each column of heatmaps represents the 4 sets of experiments. The confusion matrix shows improvement in classification based on pooled motif embedding compared to pooled gene proximity.

We also performed graph autoencoder analysis in the transcriptional microenvironments. The patches were connected to their 2 nearest neighboring patches in the same microenvironment to form the graph. Four different autoencoders were trained for the 4 sets of experiments. We compared embedded features between cell type and cell proliferation, and found several features in all sets to be significantly different (Figs. S19 and S20). Therefore, we performed the same CCC motif analysis as previously in MSCs. We extracted CCC motifs using the same GNNExplainer algorithm as the previous analysis and performed classification analysis based on the cell type and proliferation. The 5 patches closest to the immune cells were detected, and the portion of the patch nearest neighbor graph that influenced the embedding values of the proximal patch was found using GNNExplainer (Fig. 8A). The nodes with high importance and any edges that connect between these nodes were identified as a CCC motif (Fig. 8B). The pairwise gene proximity that changes the embedding results was also studied. CD45–COL1A1, IL-6–COL1A1, and ACTb–COL1A1 are the gene pairs with the highest influence on the embedding (Fig. 8C). The motifs were then projected into the latent space, and the projected features were then average pooled as the features of the CCC motif. The gene proximity of the CCC motif was also average pooled as another set of features of the CCC motif.

The pooled embedding and pooled gene proximity were used to perform t-distributed stochastic neighbor embedding (tSNE) analysis and classification into CD4^+^ or CD8^+^ T cells or KI67^+^ or KI67^−^ cells. Four different gene proximity and motif embedding tSNE analyses were performed, as the 4 sets of experiments include different genes (Fig. 8D). CD4^+^ and CD8^+^ T cell motifs show separation on the tSNE plot based on both the pooled gene proximity and pooled embedding. However, the experiment shows a low number of KI67^+^ T cells. We then performed classification analyses of the immune cell type based on the pooled CCC motif proximity and embedding. We labeled each motif based on their associated immune cell type and KI67 expression levels as 4 classes: CD4^+^_KI67^+^, CD4^+^_KI67^−^, CD8^+^_KI67^+^, and CD8^+^_KI67^−^. The data were then separated into training and testing sets with an 80%–20% split. The classification was performed using an SVM. The classification performance was measured using confusion matrices and visualized in heatmaps (Fig. 8E). The top row of the confusion matrix corresponds to the classification using pooled embedding, and the bottom row corresponds to the pooled motif gene proximity. Each column corresponds to a set of multiplexed RNA-FISH using each set of genes (Table S1). The classification based on motif gene proximity outperforms using the pooled embedding. The classification performance comparison matches the CCC motif classification comparison in MSC in hydrogel. The comparison results show that the combination of CCC motif detection using GNN explainability analysis and gene proximity can improve the classification of CCC between different cell types.

### 3D-spaGNNG-E discovers regional-specific subcellular gene localization in astrocyte–neuron CCC in mouse brain

Astrocytes play a critical role in maintaining overall health in the central nervous system. CCC between astrocytes and endothelial cells is essential for maintaining the blood–brain barrier and controlling nutrient transport into the brain [58]. Further, astrocytes and neuron interactions have been shown to regulate functions such as sleep and microcirculation [59,60]. Therefore, the central nervous system has been the subject of many studies. Recent advancements in FISH technologies have also generated large-scale 3D data in tissue. For example, the expansion-assisted iterative-FISH (EASI-FISH) protocol utilizes expansion microscopy and light sheet imaging to acquire 3D spatial transcriptomics data at single-transcript resolution [6]. MERFISH data in mouse brain sections of up to 200 μm have also been published [5]. Thus, we seek to analyze the CCC between astrocytes and neurons in the brain using the published brain MERFISH data. Previous studies have shown regional specificity of gene expression and CCC pattern [61]. Therefore, we seek to compare astrocyte–neuron CCC in the hypothalamus and cortex.

**Fig. 9.**
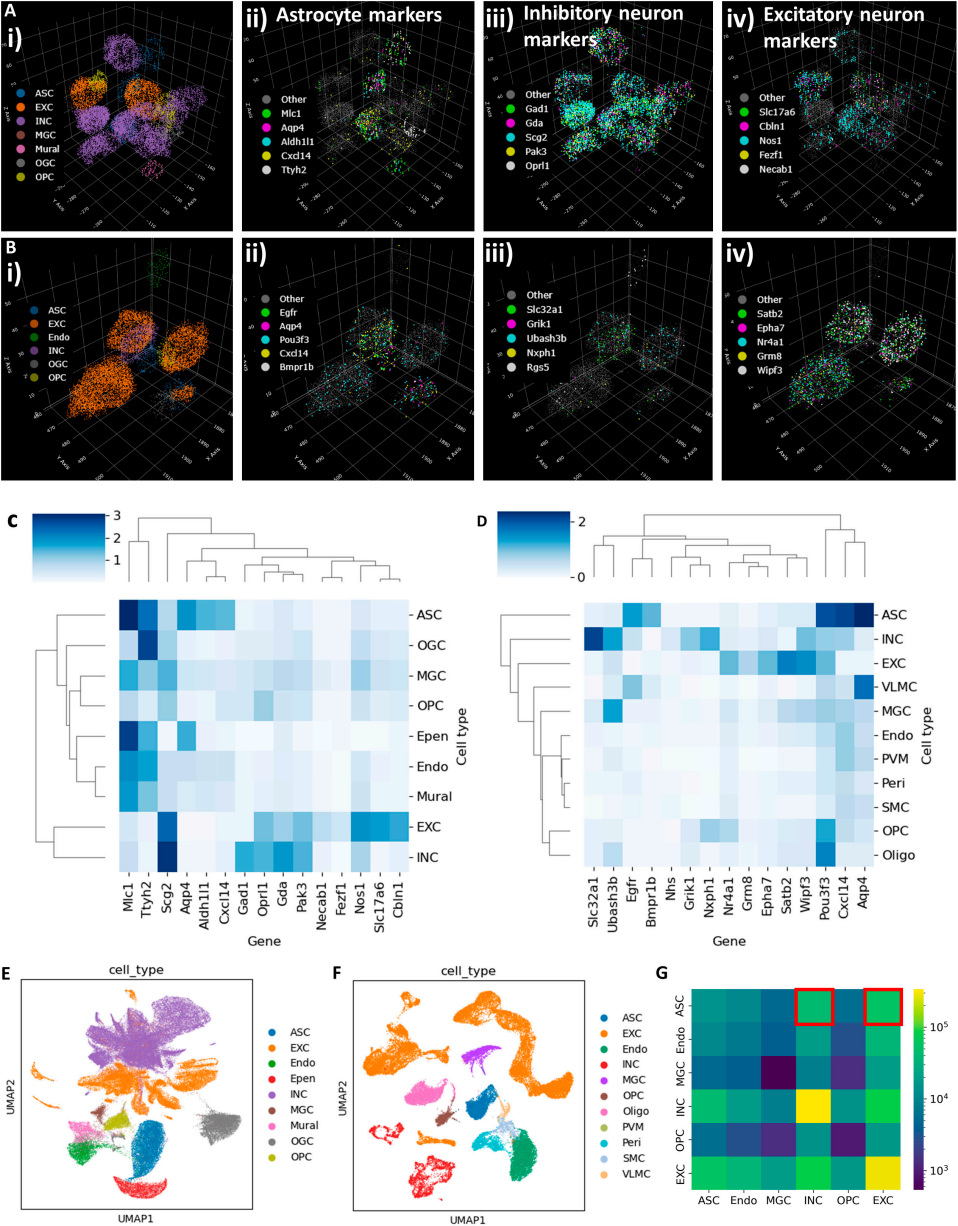
3D MERFISH data in mouse brain. (A) MERFISH data in the hypothalamus. The data contain the transcript location of 156 genes, segmentation of each cell, expression of each cell, and cell type labels. The transcripts of cells around a central astrocyte are shown. The transcripts are colored based on the cell types in (i). Astrocyte markers, inhibitory neuron markers, and excitatory neuron markers are highlighted in (ii), (iii), and (iv). (B) MERFISH data in the cortex. The data contain the transcript location of 238 genes, segmentation of each cell, expression of each cell, and cell type labels. The transcripts of cells around a central astrocyte are shown. The transcripts are colored based on the cell types in (i). Astrocyte markers, inhibitory neuron markers, and excitatory neuron markers are highlighted in (ii), (iii), and (iv). (C) Heatmap of log expression of astrocyte, inhibitory neuron, and excitatory neuron marker genes in each cell type identified in the hypothalamus MERFISH data. (D) Heatmap of log expression of astrocyte, inhibitory neuron, and excitatory neuron marker genes in each cell type identified in the cortex MERFISH data. (E) Cell phenotyping based on single-cell gene expression in the hypothalamus. The UMAP was generated based on the RNA count of each cell and cell phenotype provided in the data. (F) Cell phenotyping based on single-cell gene expression in the cortex. The UMAP was generated based on the RNA count of each cell and cell phenotype provided in the data. (G) The number of direct cell contacts between common cell types between the hypothalamus and the cortex. We established the Delaunay triangulation among locations of each cell and identified any cell pairs connected by an edge as direct contact cells. We focus our analysis on astrocyte contact with neurons.

The dataset consists of 3D MERFISH data in a 200-μm section of the mouse hypothalamus and a 100-μm section of the mouse cortex. The original images were collected using confocal microscopes, and images were enhanced using a deep-learning-based image processing algorithm. The original images were then decoded to identify each gene (Fig. 9A and B). The dataset also contains single-cell gene expression data based on the count of each gene in single cells. We first examined the cell type labels provided by the dataset by generating heatmaps of the gene expression of each cell type (Fig. 9C and D). Cell type annotation for the hypothalamus and the cortex and the corresponding cell type shows high expression of related marker genes (Fig. 9E and F). We then performed Delaunay triangulation to identify the immediate neighbors of each cell and formed the cell neighbor graph. Figure 9G shows the number of edges in the triangulation that connect 2 cell types. We focus on the highlighted astrocyte–neuron interactions (ASC: astrocyte, INC: inhibitory neurons, EXC: excitatory neurons).

**Fig. 10.**
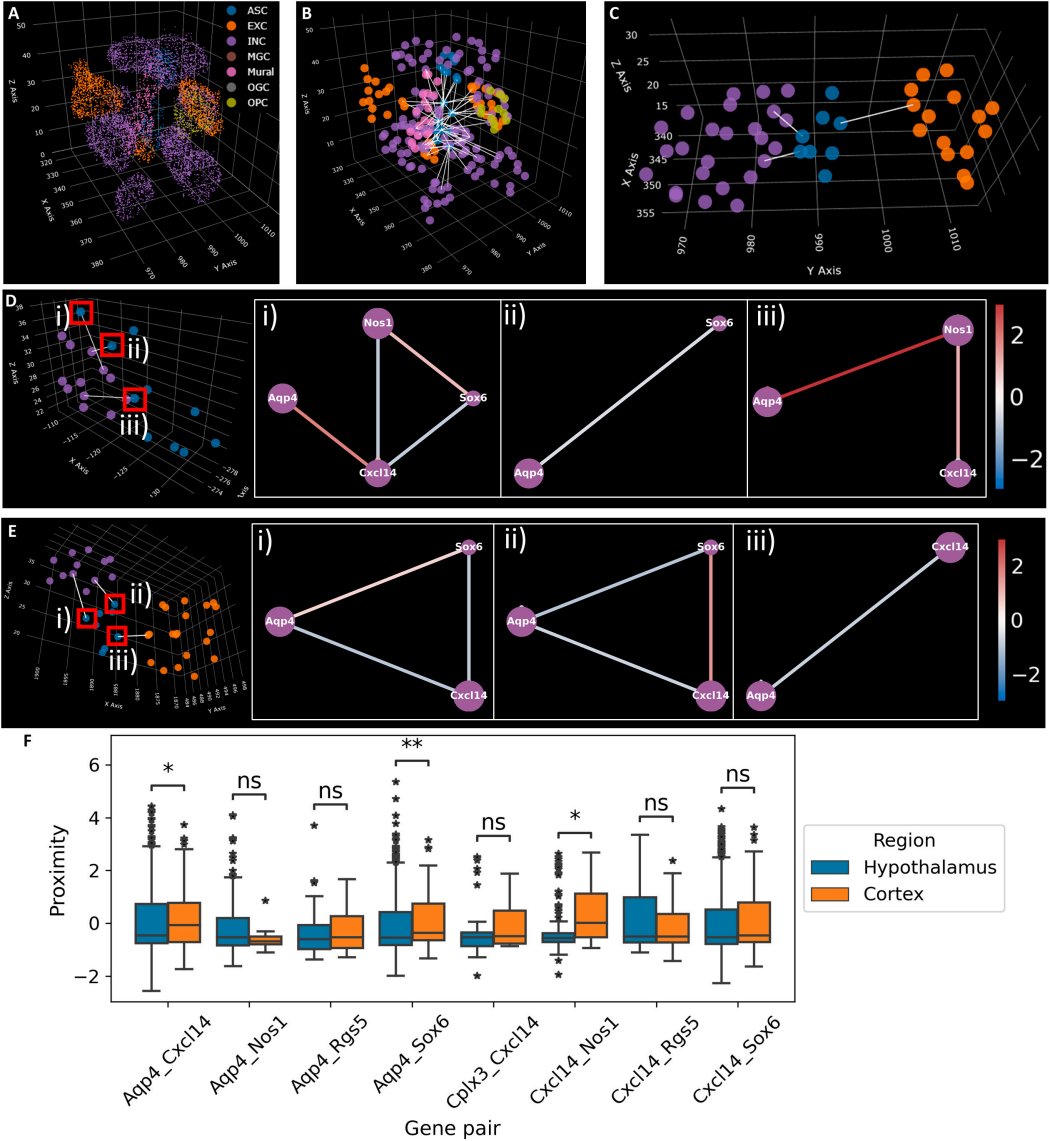
Gene proximity change in astrocyte–neuron CCC. (A) Transcripts of the astrocyte neighborhood. We established the Delaunay triangulation among all cells in the 3D MERFISH data. We then defined a central astrocyte and all cells connected to it as an astrocyte neighborhood. The scatterplot shows all transcripts in cells of an astrocyte neighborhood. Transcripts are colored according to the phenotype of the cells. (B) Subcellular patches of an astrocyte neighborhood. 3D-spaGNN-E pipeline was applied to each cell to identify subcellular patches. The scatter points show the patch center locations. We then establish the Delaunay triangulation among all patches of an astrocyte neighborhood. The edges that connect a patch of the central astrocyte and a patch of another cell are highlighted as intercellular edges. (C) CCC edges extracted from an astrocyte neighborhood. We selected the 3 shortest intercellular edges between the central astrocyte and a neuron as CCC edges. We define the astrocyte patches that are connected by the CCC edges as CCC patches. (D) Gene neighborhood network of CCC patches in the hypothalamus between selected genes. (E) Gene neighborhood network of CCC patches in the cortex between selected genes. (F) Statistical comparison between gene proximities of CCC patches in the hypothalamus and the cortex. CCC patches in the cortex show higher proximity between Aqp4-Cxcl14, Aqp4-Sox6, and Cxcl14-Nos1. The statistical significance annotation is as follows: *P* value annotation legend: ns: 5.00 × 10^−2^ < *P* ≤ 1.00, *: 1.00 × 10^−2^ < *P* ≤ 5.00 × 10^−2^, **: 1.00 × 10^−3^ < *P* ≤ 1.00 × 10^−2^, ***: 1.00 × 10^−4^ < *P* ≤ 1.00 × 10^−3^, ****: *P* ≤ 1.00 × 10^−4^.

We then identified the cell neighborhoods around each astrocyte for detailed analysis. The cell neighborhood around an astrocyte is defined as the central astrocyte and all one-hop neighbors of the astrocyte in the cell neighbor graph. The detected transcripts of an astrocyte neighborhood are shown in Fig. 10A. The spaGNN pipeline was performed on all cells in the neighborhood, and the center position of each subcellular patch was identified. We then applied the Delaunay triangulation among all patch centers in an astrocyte neighborhood. We then defined the edges between the patches of the central astrocyte and other cells as intercellular patches (Fig. 10B). We then selected the 3 shortest intercellular edges (Fig. 10C) and filtered out any shortest edges that are not between astrocytes and neurons. We defined the astrocyte patches of the selected edges as interacting patches and performed gene neighborhood network analysis. The cells interacting with astrocytes, their interacting patches, and the gene neighborhood network of the interacting patches are shown for the hypothalamus (Fig. 10D) and cortex (Fig. 10E). We then compared proximity scores of interacting patches in the hypothalamus and cortex (Fig. 10F). Interacting patches in the cortex show elevated Aqp4_Cxcl14, Aqp4_Sox6, and Cxcl14_Nos1 proximity, showing potential regional differences in astrocyte–neuron interaction. The difference can be attributed to the heterogeneity of astrocyte–neuron interactions [62]. Differences in interacting patch gene proximities could indicate that different brain region is dominated by different interactions.

## Discussion

The experimental method allows cells to be cultured in in vivo-mimicking environments. Such methods have been used to study complex physiology, such as immune reactions [63]. Gel embedding also allows cells to be cultured in microfluidic devices to study more complex physiological structures, such as the cardiovascular system [64]. Cell therapy applications can also benefit from such a method. Cell therapy can rely on critical CCC. For example, appropriate differentiation of MSCs for regenerative cell therapies relies on CCC [65]. In cancer immune therapy, the interactions between immune cells and the cancer cells are critical. Further, such systems can be used to identify key CCC abnormalities in diseases [66,67]. These systems allow multiple cells to be cultured in an in vivo*-*mimicking environment. While the CCC captured in the tissue sections is more accurate, difficulties such as sample availability remain. The flexibility of cell culture compared to tissue sections allows the cell interactions to be captured and better dissected.

We selected a widefield fluorescence microscope as our imaging method. The imaging system needs to provide enough excitation light to penetrate the samples while also being sensitive to capture the fluorescence signal deeper inside the sample. The imaging depth was limited by both the light absorption by the hydrogel material and the working distance of the high-power objective lens. Further, the autofluorescence of more complex samples, such as tissue blocks, may require other imaging technologies, such as confocal [5] and light sheet imaging [6]. Post-processing can also improve image quality [5]. However, all imaging methods suffer from low axial resolution. The resolution in the lateral direction is limited by the diffraction limit. However, the axial resolution is limited by the movement of the *z* axis of the microscope.

Through this study, we expanded the spaGNN pipeline to 3D subcellular spatial transcriptomics data. The expanded 3D-spaGNN-E pipeline utilizes single-transcript detection in 3D space to reveal local gene proximity changes. In this study, we hypothesized that the CCC between MSCs of the same subtype is different from the CCC between MSCs of different subtypes. Therefore, we performed a comparison between homotypic and heterotypic cell pairs. However, the CCC between MSCs can be more complicated than homotypic or heterotypic. Further, the CCC can occur at a range of distances. In both the MSC–MSC communication and MSC–immune communications that we analyzed, we focused on the CCC at short distances within 10 μm. Microfluidic chips have been created to isolate the effect of long- and short-distance CCC [68]. Cells can be trapped in biomaterial droplets to isolate short-distance CCC [69,70]. Cells can also be forced to remain separate in microfluidic devices to isolate long-distance CCC [71]. The combination of 3D-spaGNN-E and microfluidic devices designed to isolate CCC distance could help study the gene proximity changes of CCC at varying distances.

We selected a convolutional encoder to embed the node features, and we trained the embedding to predict edges using the embedding features. Previous algorithms have been proposed to perform similar tasks. Traditional methods such as DeepWalk, node2vec, and LINE utilize techniques such as search and random walks to generate neighborhoods from a source node. The algorithms then optimize an objective function that is designed to predict graph features over an embedding function [72–74]. These methods are less flexible when faced with the diverse node features of the subcellular patches. Alternatively, more complex deep learning structures, such as attention and aggregation mechanisms, can better embed the nodes by considering the heterogeneity in the graph [75]. However, the convolutional structure provides better scalability, given the large number of features per node.

In both the MSC–MSC and MSC–immune communications, we discovered that the autoencoder embedding of the patch nearest neighbor graph changes the CCC discovery. In the MSC–MSC communications, the embedding of the CCC motif changes the prediction of homotypic and heterotypic CCC patches. In the MSC–immune communications, more embedding features of the CCC border patches show statistical significance between CD4^+^ and CD8^+^ T cells. The differences show that the message passing-based convolutional encoder combines the gene proximity of surrounding patches to the border patches and introduces the changes in transcript spatial proximity of the surrounding patches into the border patches. We utilized classification from the same algorithm based on gene proximity and motif embedding as the indicator of correlation between a set of features and potential CCC sites. However, the classification performance can also be biased as the dataset is imbalanced. For example, in the MSC–immune CCC comparison, the low number of KI67^+^ cells can hinder the classification algorithm’s performance.

The autoencoder analysis was not performed in the mouse brain 3D MERFISH data. The autoencoder is trained to predict the edges between any pair of nodes. This method requires the graph to be sparse, thus providing enough node pairs that are not connected to be used as negative labels. We elect to construct nearest neighbor graphs among all patches for each cell. Therefore, more nodes are required. In the 3D MERFISH in mouse brain dataset, the cortex and the hypothalamus studies were labeled with different sets of genes. Utilizing all genes provides enough nodes to construct sparse nearest neighbor graphs, but embedded features could not be directly compared between the hypothalamus and cortex data. We could not find enough nodes to form a sparse nearest neighbor graph when only the common genes were selected.

Many methods have been developed to infer CCC from transcriptomics data. However, most methods focus on finding changes in the expression of ligand–receptor pairs. Such methods do not consider the spatial limit of CCC. The subcellular transcript localization is also ignored. The 3D-spaGNN-E finds potential CCC sites using subcellular gene proximity. The 3D-spaGNN-E pipeline provides a data-driven approach that bases potential CCC sites on spatial information. However, the algorithm only provides an inference based on the assumption that CCC happens within a short distance, and the effect of CCC reduces as the distance between cells increases. Further, the autoencoder of 3D-spaGNN-E seeks to reconstruct the subcellular graph representation of the transcriptomics data. Such methods require a sparse graph with enough nodes without an edge connecting them. This requires the original data to contain enough detected transcripts. Otherwise, the autoencoder cannot be trained.

New methods such as SCS [76] and BIDCell [77] provide biology-informed image processing of subcellular spatial transcriptomics data. We performed cell segmentation using Cellpose [78] and then detected the transcripts. The analysis results were then used for downstream analysis. Our approach only requires the original image and does not rely on additional data from similar samples. This allows us to avoid the batch differences between the study samples and the samples in existing data. The summarized results also reduce the data size. While the original image provides more information, the majority of the pixels in the original images are dark and carry little information. Performing analysis using the detected position of transcripts also helps us to expand our method to additional datasets.

## Conclusion

We examined cells cultured in 3D environments and tissues. Using the multiplexed RNA-FISH technique, we studied the subcellular transcript distribution in MSCs in 3D hydrogel. We first applied the 3D-spaGNN-E pipeline to study the pairwise gene proximity relationships. We further expanded the pipeline to incorporate graph autoencoder to reduce the number of features of each node while convolving the gene proximities of neighboring patches. The sample contains cells located in close proximity; thus, we examined the potential cell–cell interactions through gene proximity and graph autoencoder embedding. Both local gene proximity and autoencoder embedding show changes between sites of different cell interactions. We then utilized Cultrex gel to coculture MSCs and PBMCs to study MSC–immune interactions. Gene proximity can reveal potential MSC–immune interactions, while the autoencoder embedding of the patch graph can potentially improve the cell interaction prediction.

## Methods

### MSC 3D hydrogel assembly

The hydrogel containing 6% 4 arms PEG-MAL was synthesized using PEG-MAL macromer, adhesive peptides, and crosslinkers. PEG-MAL macromers were tethered with a 1:4 ratio of macromers and thiolated GFOGER peptide at a pH of 7.8 Hepes by incubating them for 30 min at 37 °C. All components were diluted using phosphate-buffered saline, CaCl_2_^+^, MgCl_2_^+^ (PBS^++^) solution with a pH of 7.4 and 1% Hepes. Covalent conjugation ingredients, a 1:1 mixture of matrix metalloproteinase-9 (MMP-9) degradable VPM peptide to nondegradable dithiothreitol (DTT) molar ratio, were suspended together at a pH of 6.4 Hepes buffer. Cells (UC and BM-MSCs) were dissolved in the crosslinker solution. Hydrogels were crosslinked by a mixture of a 1:1 volume ratio of macromers and crosslinker–cell solutions on the nontreated plate surface. The mixture was mixed rapidly via pipet and cured at 37 °C for 15 min. Post-curing, the cell media, or buffers, were added to each hydrogel assembly. The medium was replenished every 3 d. To assist imaging, the hydrogel is prepared in 96-well plates (catalog #265300).

### 3D hydrogel compounds

4 Arms (20 kDa, ≥90% purity) and DTT were purchased from Laysan Bio and Sigma-Aldrich, respectively. MMP-9 degradable VPM peptide (GCRDVPMSMRGGDRCG, >90% purity) and collagen-mimetic GFOGER peptide [GYGGGP(GPP)5GFOGER(GPP)5GPC, >90% purity] were purchased from AAPPTec (Louisville, KY). All ingredients were reconstituted in 0.01 M 4-(2-hydroxyethyl)-1-piperazineethanesulfonic acid (Hepes) buffer with a pH of 7.4.

### Cultrex-base membrane extract gel coculture of MSCs and PBMCs

After expansion in tissue culture flasks and passaging, patient-derived BM-MSCs were first cultured on poly-l-lysine-coated glass coverslips for 24 h to allow adherence. The cells were then primed in culture medium with 50 ng/ml of IFN-γ for 24 h. At the same time, PBMCs isolated from whole blood and stored in liquid nitrogen were thawed and cultured in a tissue culture flask. At the end of priming time, PBMCs were centrifuged and resuspended in serum-free RPMI 1640 medium. The cell suspension was then chilled on ice. Once the cell suspension was cold, the thawed Cultrex gel was mixed with cell suspension at 20%. The glass coverslips with MSCs were washed with 1× PBS. Fifty microliters of cell suspension and Cultrex gel mix was then placed on top of the coverslips and incubated at 37 °C for 1 h. After 1 h, the T cell medium supplemented with phorbol 12-myristate 13-acetate (PMA) and ionomycin were added and cultured for 3 d before fixation and labeling.

### Multiplexed RNA detection

We performed the third-generation HCR for RNA-FISH following the HCR mammalian cells on slide protocol provided by Molecular Instruments. The samples were first fixed with 4% paraformaldehyde (10 ml of 16% paraformaldehyde, 4 ml of 10× PBS, 26 ml of ultrapure water for 40 ml of fixation buffer) at room temperature for 15 min and washed with 1× PBS. The samples were then permeabilized in 70% ethanol at −20 °C for over 12 h.

After permeabilization, the samples were air-dried for 10 min and washed with 2× saline sodium citrate (SSC) buffer 3 times. The samples were then incubated in a 300-μl pre-warmed HCR probe hybridization buffer (lot #BPH02023) at 37 °C for 30 min for prehybridization. HCR probes (3 μl) for desired targets were then added to 300 μl of pre-warmed probe hybridization buffer. The prehybridization buffer was aspirated, and the probe dilute was added to the samples. The samples were incubated at 37 °C for 12 to 16 h. The samples were then washed with 300 μl of pre-warmed probe wash buffer (lot #BPW02922) for 5 min at 37 °C 4 times. Then, the samples were rinsed with 300 μl of 5× saline sodium citrate with Tween 20 (SSCT) for 5 min at room temperature twice. The samples were then incubated with 300 μl of amplification buffer (lot #BAM02422) at room temperature for 30 min for pre-amplification. Six microliters of each HCR amplifier hairpin was then snap-cooled to 95 °C for 90 s and cooled down to room temperature in a dark drawer for 30 min. The snap-cooled hairpins were then added to 300 μl of amplification buffer. The pre-amplification buffer was then aspirated, and diluted hairpins were added to the sample. The samples were then incubated for 1 h and 15 min. The amplification solution was then aspirated, and the samples were washed with 5× SSCT for 5 min at room temperature 5 times (see HCR protocol for cells on slide).

The samples were then mounted in an antifade mounting buffer containing Tris-HCl (20 mM), NaCl (50 mM), glucose (0.8%), saturated Trolox (Sigma, 53188-07-1), pyranose oxidase (Sigma, P4234), and catalase (Sigma, 9001-05-2, 1:1,000 dilution).

The imaging was performed using a widefield fluorescence microscope. A Z-step of 0.3 was selected to achieve sufficient detection of RNA transcripts.

To remove fluorescent mRNA signals, we used ribonuclease (RNase)-free deoxyribonuclease (DNase) I (Sigma, 04716728001). After imaging, the samples were first washed with 2× SSC twice. We then diluted 50 μl of 10× concentration incubation buffer in 450 μl of RNase-free water to 1× concentration. The samples were then incubated with a 1× incubation buffer at room temperature for 5 min. Then, 10 μl of DNase I, 50 μl of 10× incubation buffer, and 440 μl of RNase-free water were mixed. The samples were then incubated in the DNase I mixture for 4 h at room temperature. After incubation, the samples were then washed with 30% formamide in 2× SSC at room temperature for 5 min 3 times. Removal of signals was confirmed under a fluorescent imaging microscope. The samples were then ready to start the next cycle of RNA labeling.

### Graph autoencoder

After the subcellular gene neighborhood network is calculated, a patch nearest neighbor graph was generated by connecting an edge between a patch and its 2 nearest patches. The pairwise gene proximity is used as the node features. The patch nearest neighbor graph of each cell was then combined to form a single graph by concatenating node features and edges. The graph autoencoder takes 2 matrices as input. The first is a node feature matrix X and an edge matrix E. The feature matrix has the shape of $N_{\text{nodes}}\times M_{\text{gene pairs}}$. The edge matrix is a sparse representation of the adjacency matrix with the shape of $2\times n_{\text{edges}}$, where each column contains the indices of the nodes connected by the edge.

The combined graph was then separated into training and testing sets. Twenty percent of the edges were used as the testing set, and 10% of the edges were used as the validation set. The remaining edges were used as the training set. The graph autoencoder encodes the nodes using 2 convolutional layers and a single dropout layer that randomly drops out 50% of the features. The inner product computes the probability that an edge exists between 2 nodes based on the following formula:

$$p_{ij}=-\;\frac{1}{1-e^{<z_{1},z_{2}>}}$$ (1)

where $p_{ij}$ is the predicted probability that an edge exists between ${\text{node}}_{i}$ and ${\text{node}}_{j}$, and $z_{i}$ and $z_{j}$ are embedded features of ${\text{node}}_{i}$ and ${\text{node}}_{j}$, respectively.

After computing prediction, the following reconstruction loss function was optimized over the convolutional layer weights:

$$\begin{array}{c} L=-\;\text{log}\left(\frac{1}{n_{pos}}\sum_{pos_{ij}}^{n_{pos}}p_{ij}\right)-\text{log}\left(\frac{1}{n_{neg}}\sum_{neg_{ij}}^{n_{neg}}\left(1-p_{ij}\right)\right) \end{array}$$ (2)

where $n_{\mathrm{pos}}$ is the number of edges in the training set and $n_{\mathrm{neg}}$ is the number of node pairs that lack an edge. $\mathrm{po}s_{\mathrm{ij}}$ indicates node pairs that are connected by an edge, and $\mathrm{ne}g_{\mathrm{ij}}$ is node pairs that lack edge connection.

### Statistics

We applied a Mann–Whitney–Wilcoxon test to calculate *P* values in Fig. 7A and B, and the α value is corrected by Bonferroni correction. The statistical comparison in Fig. 7C and D was done using the Mann–Wilcoxon test and corrected by Bonferroni correction.

## Data Availability

Code generated in this study is available at: https://github.com/coskunlab/3D-spaGNN-E. Data generated in this study are available at: https://figshare.com/articles/dataset/Data_for_the_manuscript_Graph-based_3D_spatial_gene_neighborhood_networks_in_gel-embedded_single_cells/25962691.
