# Supplementary material for: Graph-Based 3-Dimensional Spatial Gene Neighborhood Networks of Single Cells in Gels and Tissues
Source: BME Front. 2025 Mar 13;6:0110. doi: 10.34133/bmef.0110 (PMC11906096; doi:10.34133/bmef.0110)
Supplement: Supplementary 1 — Figs. S1 to S24 Table S1 [file bmef.0110.f1.pdf]

Supplemental Information

Graph-based 3D spatial gene neighborhood networks of single cells in gels and tissues

Zhou Fang[1,2], Kelsey Krusen[1], Hannah Priest[1], Mingshuang Wang[1], Sungwoong Kim[1], Anirudh Sriram[1], Ashritha Yellanki[1], Ankur Singh[1,3,4,5], Edwin Horwitz[6], Ahmet F. Coskun[1,4,5*]

[1] Wallace H. Coulter Department of Biomedical Engineering, Georgia Institute of Technology and Emory University, Atlanta, GA, USA

[2] Machine Learning Graduate Program, Georgia Institute of Technology, Atlanta, GA, USA

[3] Woodruff School of Mechanical Engineering, Georgia Institute of Technology, Atlanta, GA, USA

[4] Interdisciplinary Bioengineering Graduate Program, Georgia Institute of Technology, Atlanta, GA, USA

[5] Parker H. Petit Institute for Bioengineering and Bioscience, Georgia Institute of Technology, Atlanta, GA 30332

[6] Department of Pediatrics, Emory University School of Medicine, Atlanta, GA 30322

*Correspondence and lead contact: Ahmet F. Coskun, Ph.D. (ahmet.coskun@bme.gatech.edu)

Clustering into patches comparison between 2D and 3D

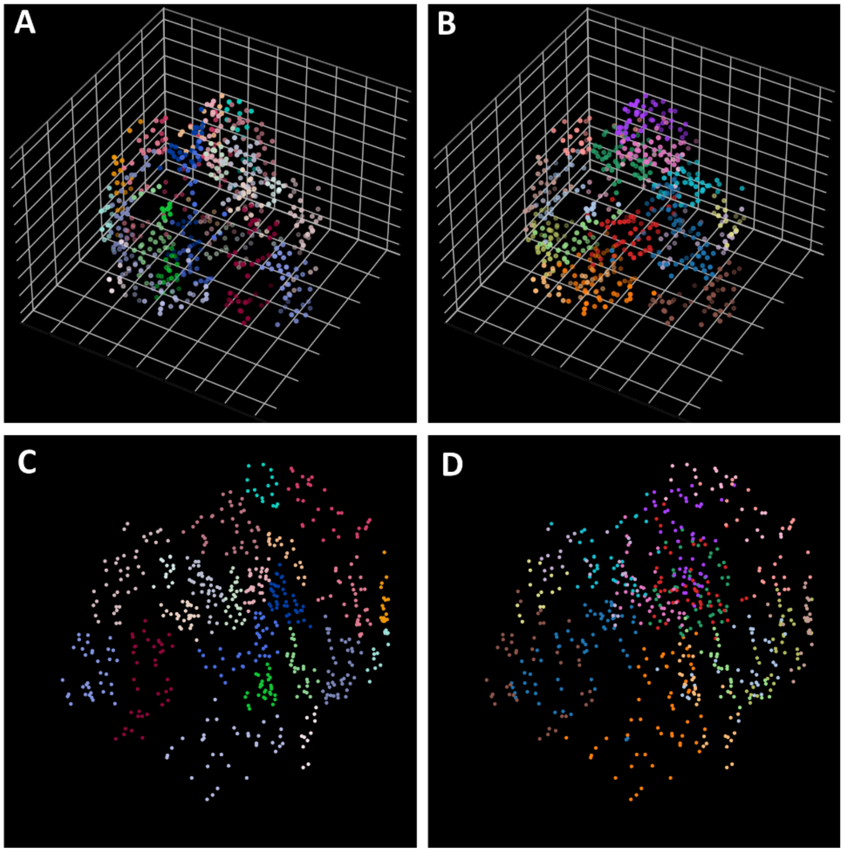

Gene nearest neighbors 2D and 3D comparison

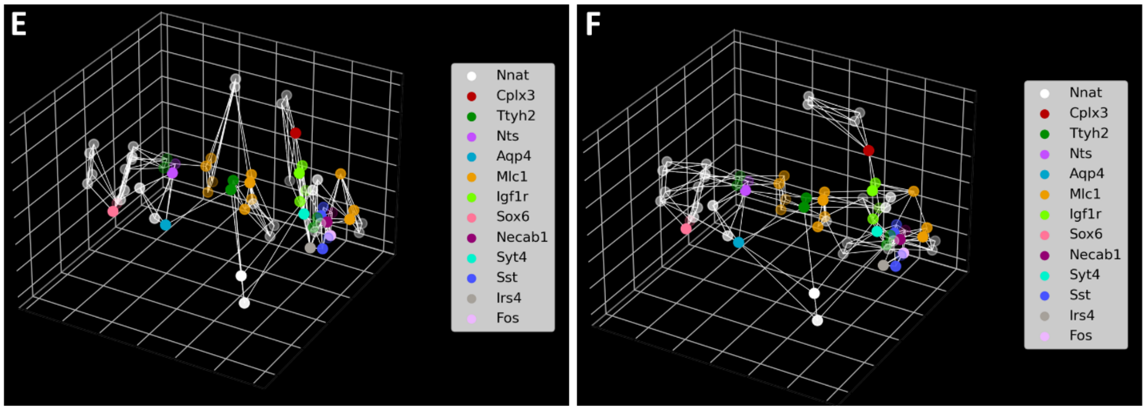

Fig. S1: 2D and 3D spaGNN comparison

A. 3D view of patching of a single cell using clustering when the cell is projected into a 2D plane.
B. 3D view of patching of a single cell using clustering using the 3D positions of transcripts.

C. Top view of patching of a single cell using clustering when the cell is projected into a 2D plane. The view shows no overlapping in XY plane.
D. Top view of patching of a single cell using clustering using the 3D positions of transcripts. The patches overlap in XY plane, but provides better patching for transcripts that are close by in XY plane but distant in Z axis.
E. Nearest neighbor graph among all transcripts of a patch using 2D projected transcript positions.
F. Nearest neighbor graph among all transcripts of a patch using 3D positions of transcripts. Nearest neighbor graph generated using 2D projected transcript positions connects transcripts separated along the Z-axis as they are close in XY plane.

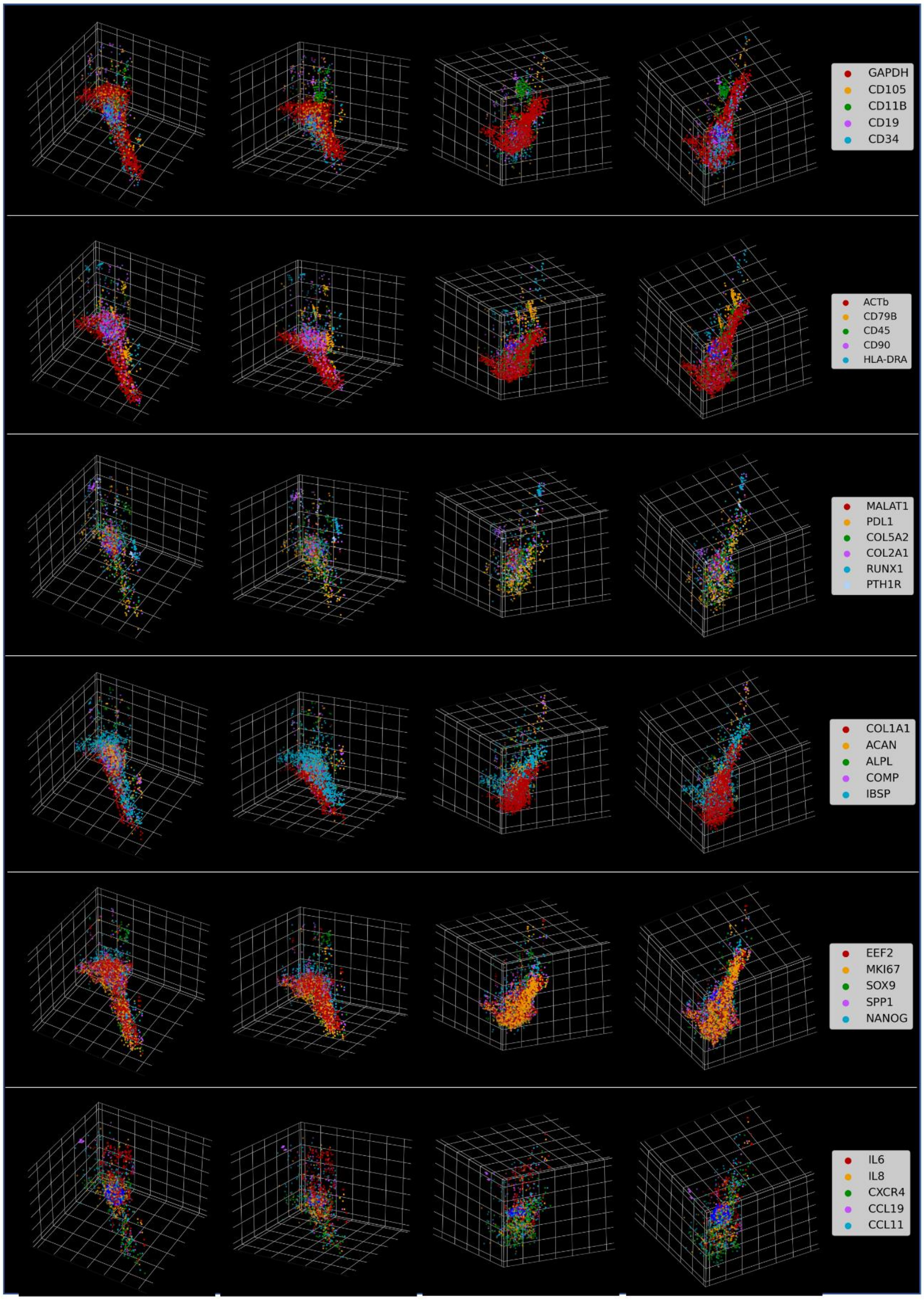

Fig. S2: Multiview of detected transcripts in the BM-MSC presented in Fig. 1B.

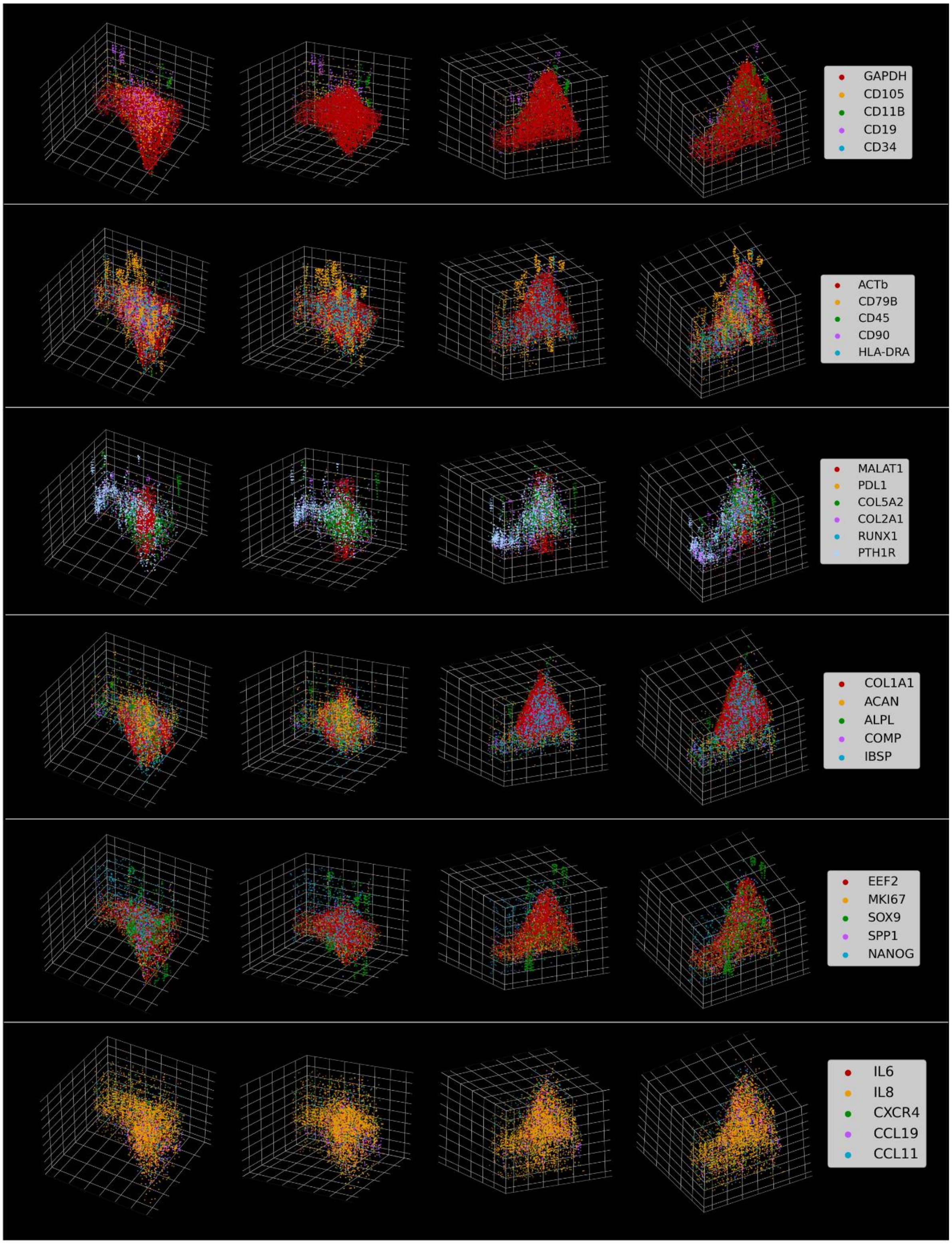

Fig. S3: Multiview of detected transcripts in the BM-MSC presented in Fig. 1B.

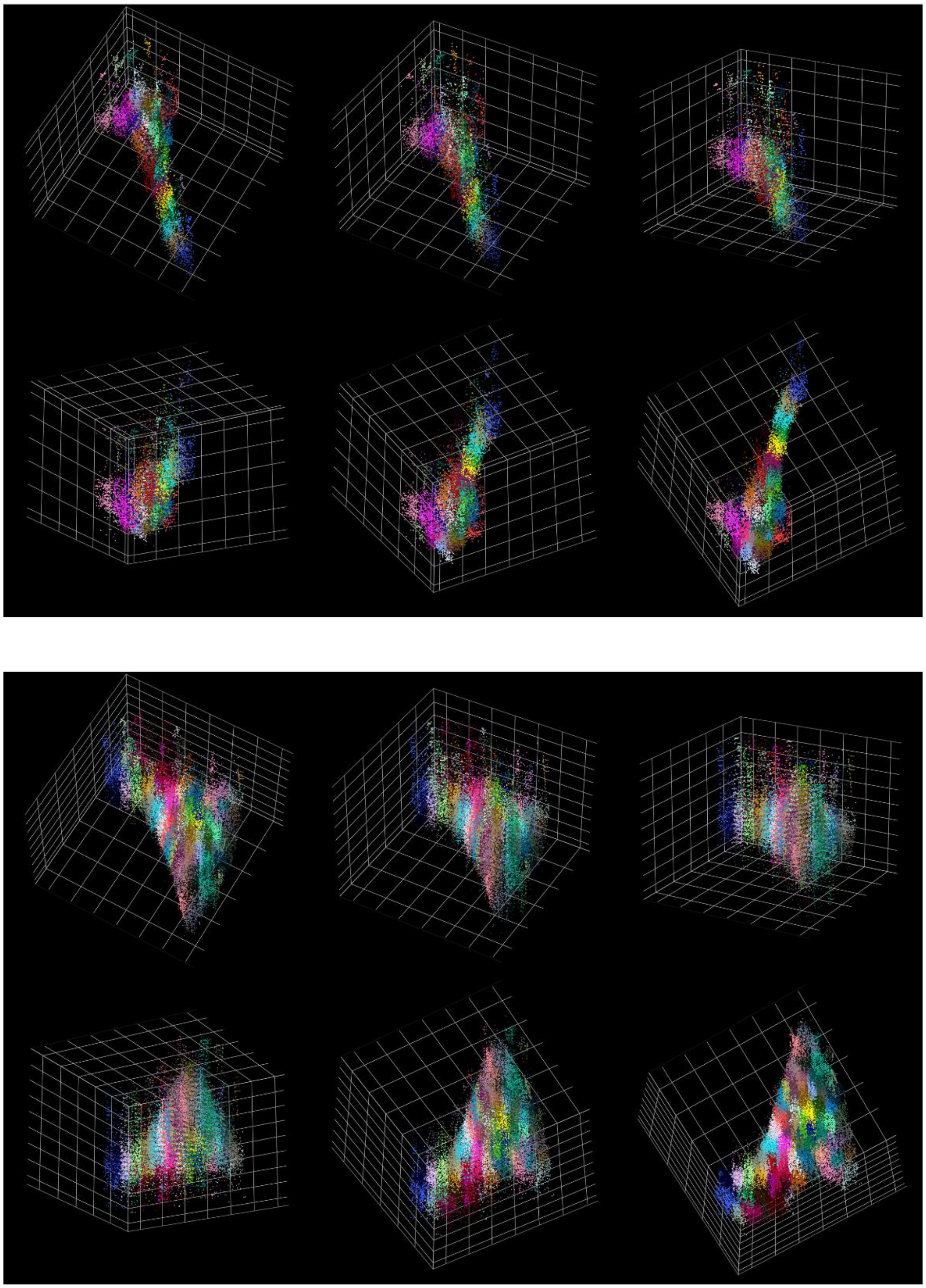

Fig. S4: Multiview of patching of the BM-MSC (top) presented in Fig. 2B and UC-MSC (bottom) presented in Fig. 2C

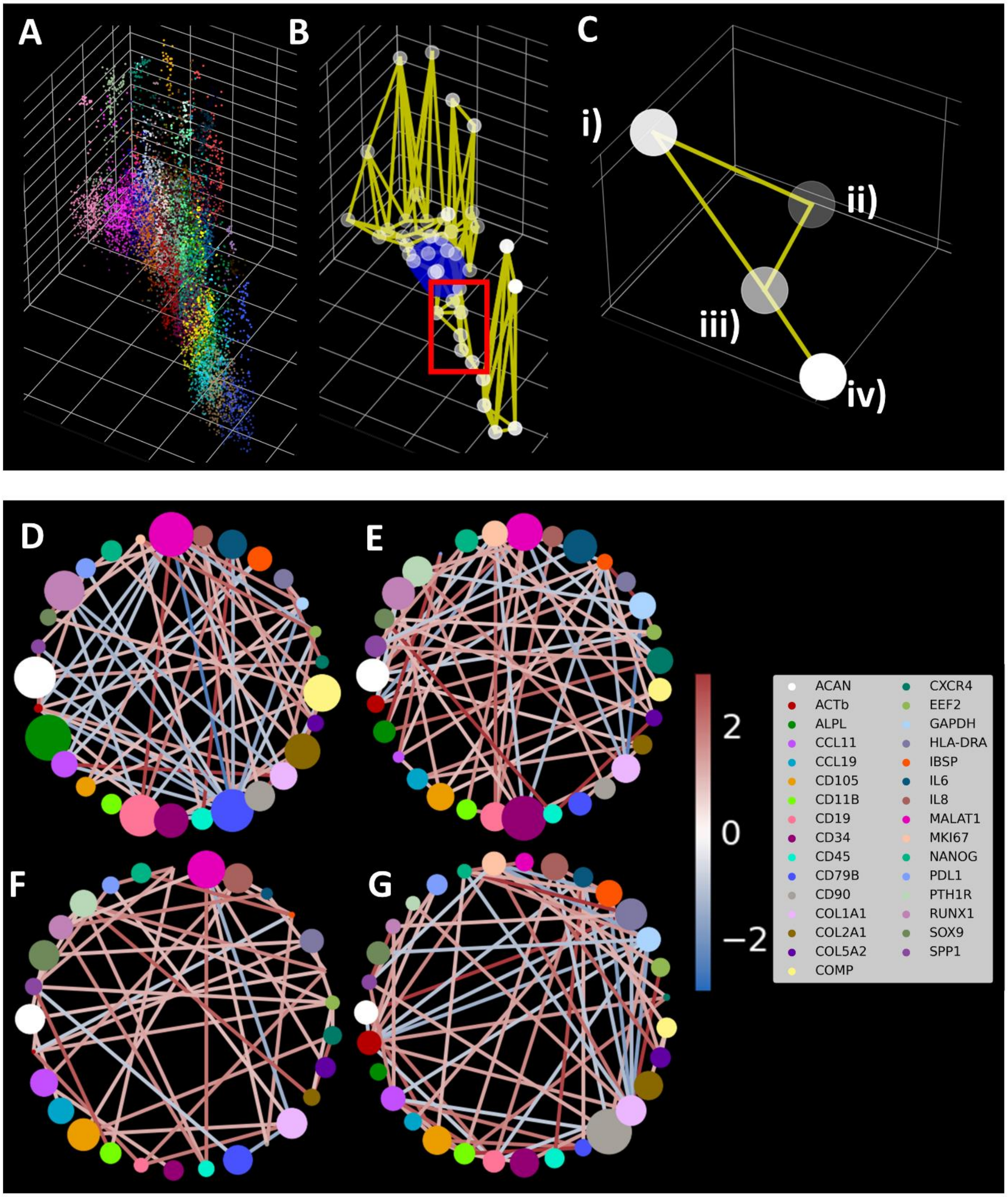
A
B
C
i)
ii)
iii)
iv)
D
E
F
G
2
0
−2
ACAN
ACTb
ALPL
CCL11
CCL19
CD105
CD11B
CD19
CD34
CD45
CD79B
CD90
COL1A1
COL2A1
COL5A2
COMP
CXCR4
EEF2
GAPDH
HLA-DRA
IBSP
IL6
IL8
MALAT1
MKI67
NANOG
PDL1
PTH1R
RUNX1
SOX9
SPP1

Fig. S5: Full gene neighborhood network of the BM-MSC presented in Fig. 2E.

A. The patch separation of a BM-MSC by applying Leiden clustering to the positions of detected RNA. The x and y axes have grid spacing of 10µm and the z-axis has grid spacing of 1µm.
B. The patch graph among all patches of the BM-MSC.
C. Subgraph of the patch graph from Fig. S5B.
D. Gene neighborhood network of patch i).
E. Gene neighborhood network of patch ii).
F. Gene neighborhood network of patch iii).
G. Gene neighborhood network of patch iv).

In gene neighborhood networks, the edge color shows the proximity score of the pair of genes following the color bar shown on the right. Each node represents a gene. The node color follows the legend shown on the right. The size of each node represents the relative enrichment of the gene in the patch.

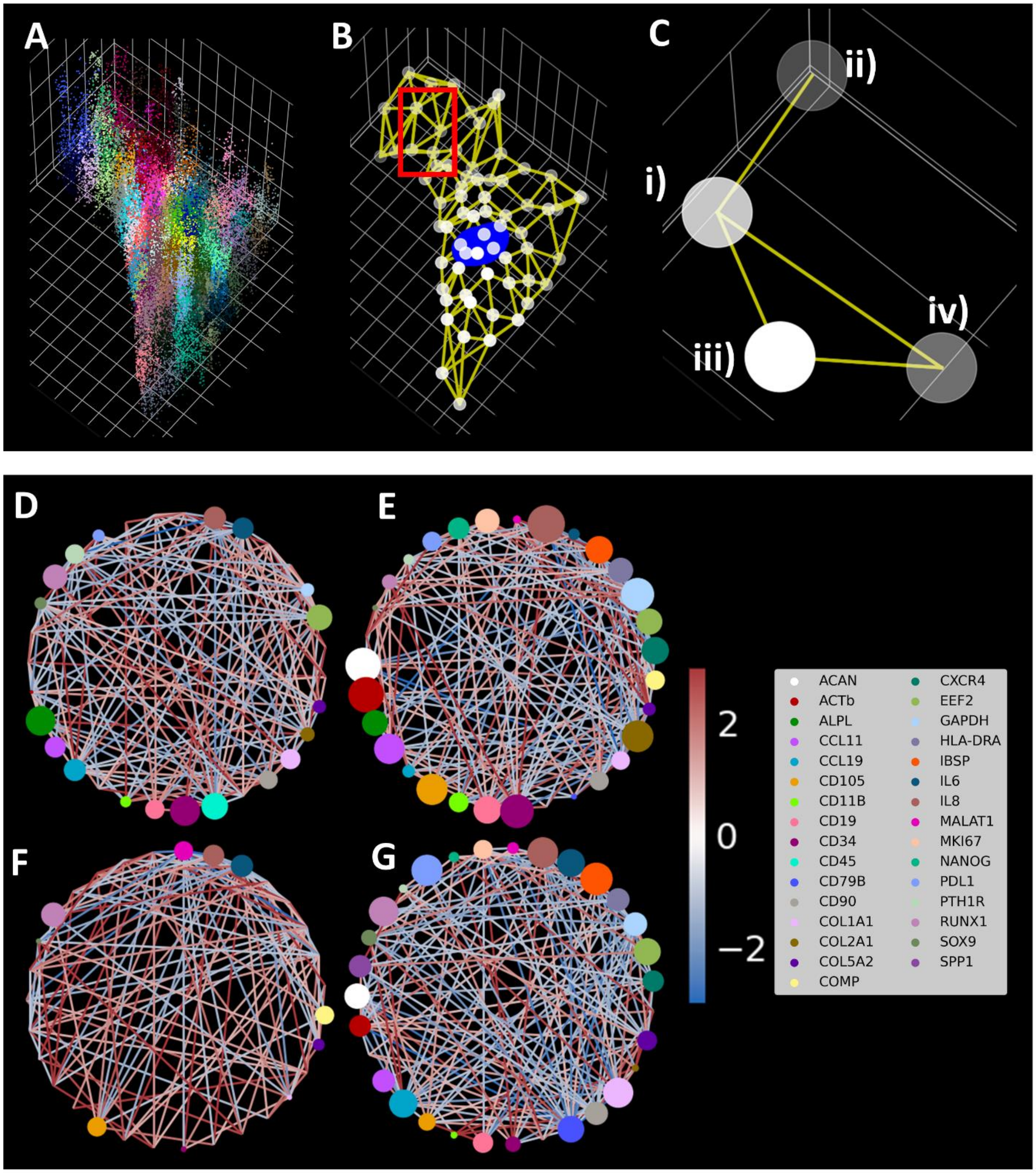
A
B
C
ii)
i)
iii)
iv)
D
E
F
G
2
0
−2
ACAN
ACTb
ALPL
CCL11
CCL19
CD105
CD11B
CD19
CD34
CD45
CD79B
CD90
COL1A1
COL2A1
COL5A2
COMP
CXCR4
EEF2
GAPDH
HLA-DRA
IBSP
IL6
IL8
MALAT1
MKI67
NANOG
PDL1
PTH1R
RUNX1
SOX9
SPP1

Fig. S6: Full gene neighborhood network of the BM-MSC presented in Fig. 2E.

A. The patch separation of a UC-MSC by applying Leiden clustering to the positions of detected RNA. The x and y axes have grid spacing of 10µm and the z-axis has grid spacing of 1µm.
B. The patch graph among all patches of the UC-MSC.
C. Subgraph of the patch graph from Fig. S6B.
D. Gene neighborhood network of patch i).
E. Gene neighborhood network of patch ii).
F. Gene neighborhood network of patch iii).
G. Gene neighborhood network of patch iv).

In gene neighborhood networks, the edge color shows the proximity score of the pair of genes following the color bar shown on the right. Each node represents a gene. The node color follows the legend shown on the right. The size of each node represents the relative enrichment of the gene in the patch.

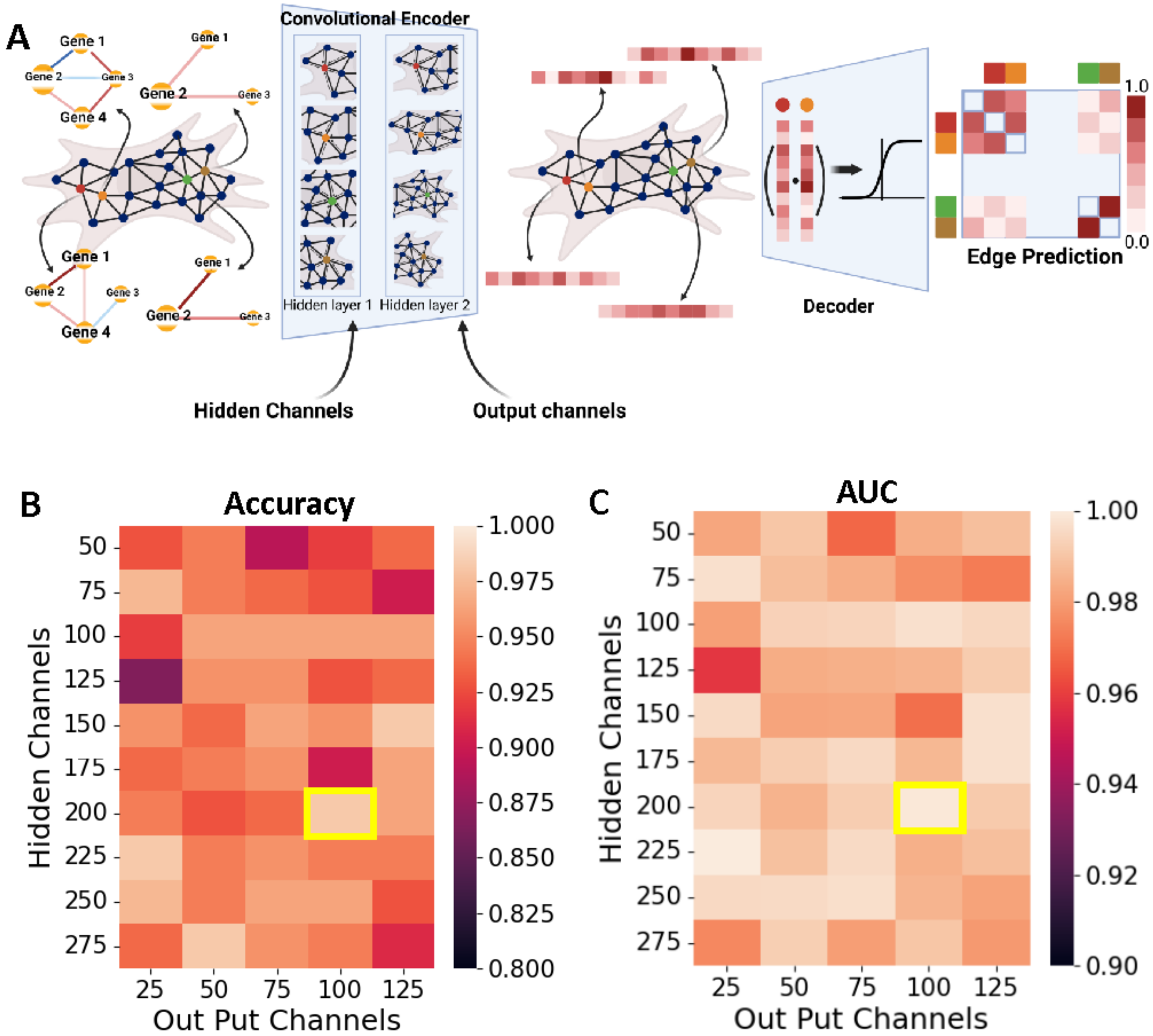

Fig. S7: Hyperparameter tuning of graph autoencoder

A. The architecture of the autoencoder. The autoencoder consists of a convolutional encoder made up of two convolution layers and a dropout layer. The number of input channels is the same as the number of original unique gene pairs. The output of the first hidden layer is the hidden channel. The output of the second layer is the output channel. Both the hidden channel and the output channel are tuned by grid search.

B. BM-MSC v. UC-MSC prediction accuracy of grid search of hyperparameters. The embedded features of subcellular patches were average-pooled by cell, and the pooled features were used to predict the cell as BM-MSC or UC-MSC.

C. BM-MSC v. UC-MSC prediction area under receiving-operating curve (AUC) of grid search of hyperparameters. A hidden channel of 200 and an output channel of 100 were selected.

A

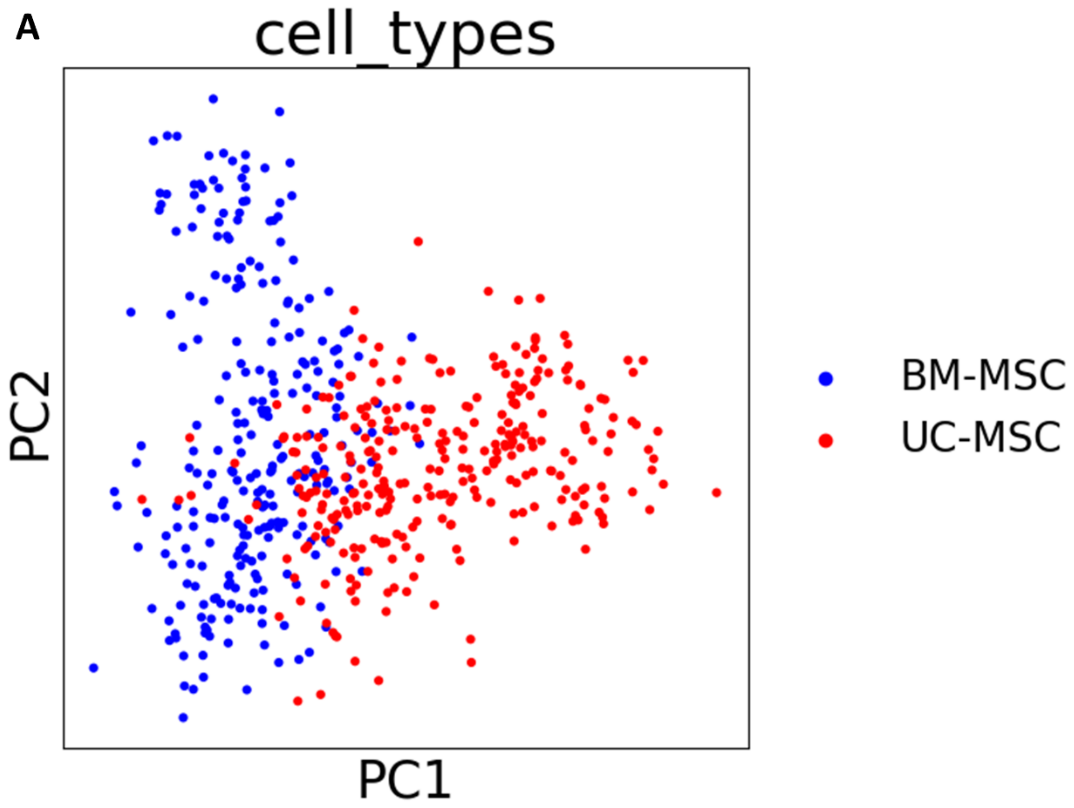
cell_types
PC2
PC1
BM-MSC
UC-MSC

B

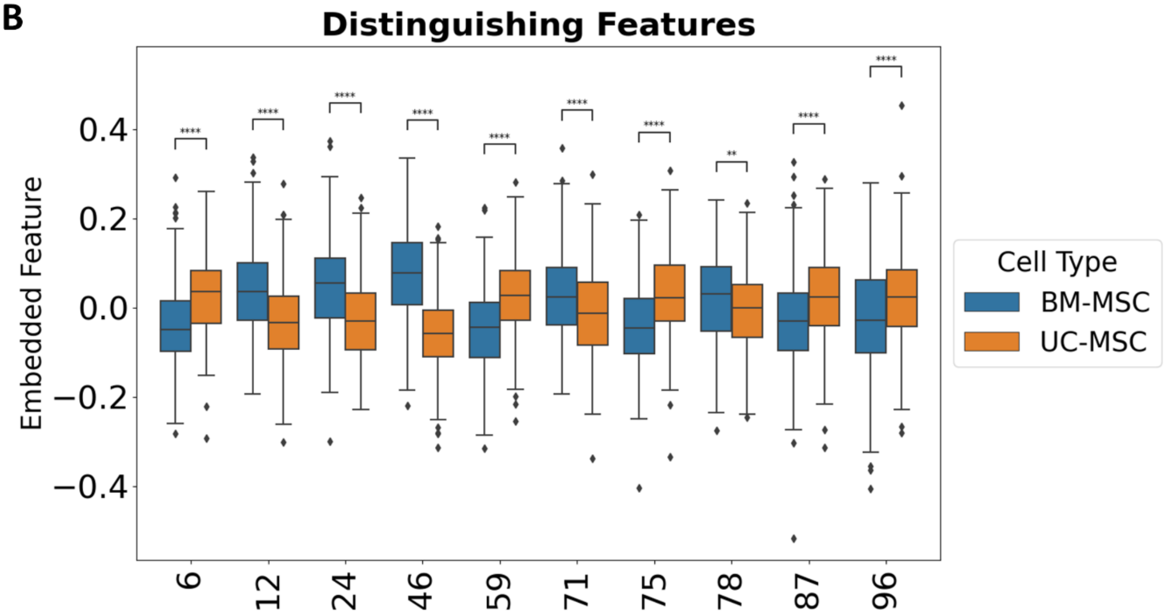
Distinguishing Features
Embedded Feature
0.4
0.2
0.0
−0.2
−0.4
6
12
24
46
59
71
75
78
87
96
Cell Type
BM-MSC
UC-MSC

Fig. S8: Principal component analysis of embedded features.

A. Scatter plot of pooled embedded features of single cells on principal components 1 and 2. After the graph autoencoder was trained, the single-cell patch nearest neighbor graphs were embedded using the encoder section. The embedded features of all nodes in the same cells were average-pooled to form a single feature vector for the cell. We then performed a principal component analysis to identify the distinguishing embedded features between BM-MSCs and UC-MSCs. The PCA analysis shows the BM-MSCs and UC-MSCs are separated along the first principal component.
B. Comparison of embedded features between BM-MSCs and UC-MSCs. By finding features that have the heaviest positive or negative bias in the first principal component, we identified the top ten distinguishing embedded features between BM-MSCs and UC-MSCs.

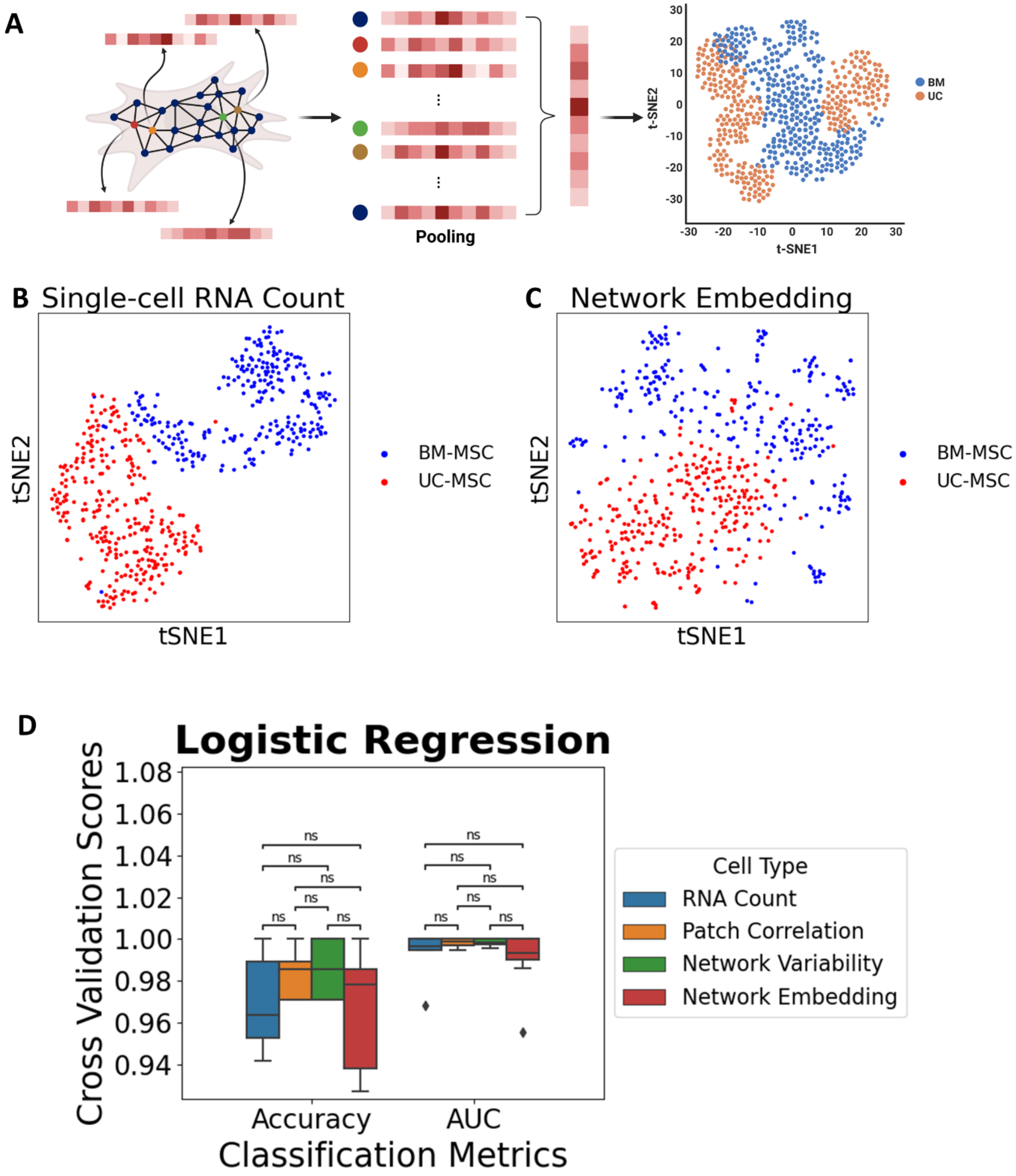
A
Pooling
t-SNE2
t-SNE1
BM
UC
B Single-cell RNA Count
tSNE2
tSNE1
BM-MSC
UC-MSC
C Network Embedding
tSNE2
tSNE1
BM-MSC
UC-MSC
D
Logistic Regression
Cross Validation Scores
ns
Accuracy
AUC
Classification Metrics
Cell Type
RNA Count
Patch Correlation
Network Variability
Network Embedding

Fig. S9: Classification of cell types using single-cell RNA count and graph autoencoder embedded features

A. Average pooling of embedded features. Features of all nodes in a cell were pooled by average to determine a set of features that describe a cell. The pooled features of each cell can be used to cluster and classify cell types.

B. tSNE plot of single-cell RNA count.

C. tSNE plot of single-cell pooled embedded features.

D. Classification performance of logistic regression when classifying BM-MSC or UC-MSC based on single-cell RNA count, patch correlation, network variability, and pooled network embedding features. All four datasets perform similarly when measured by accuracy and area under the receive-operating curve (AUC).

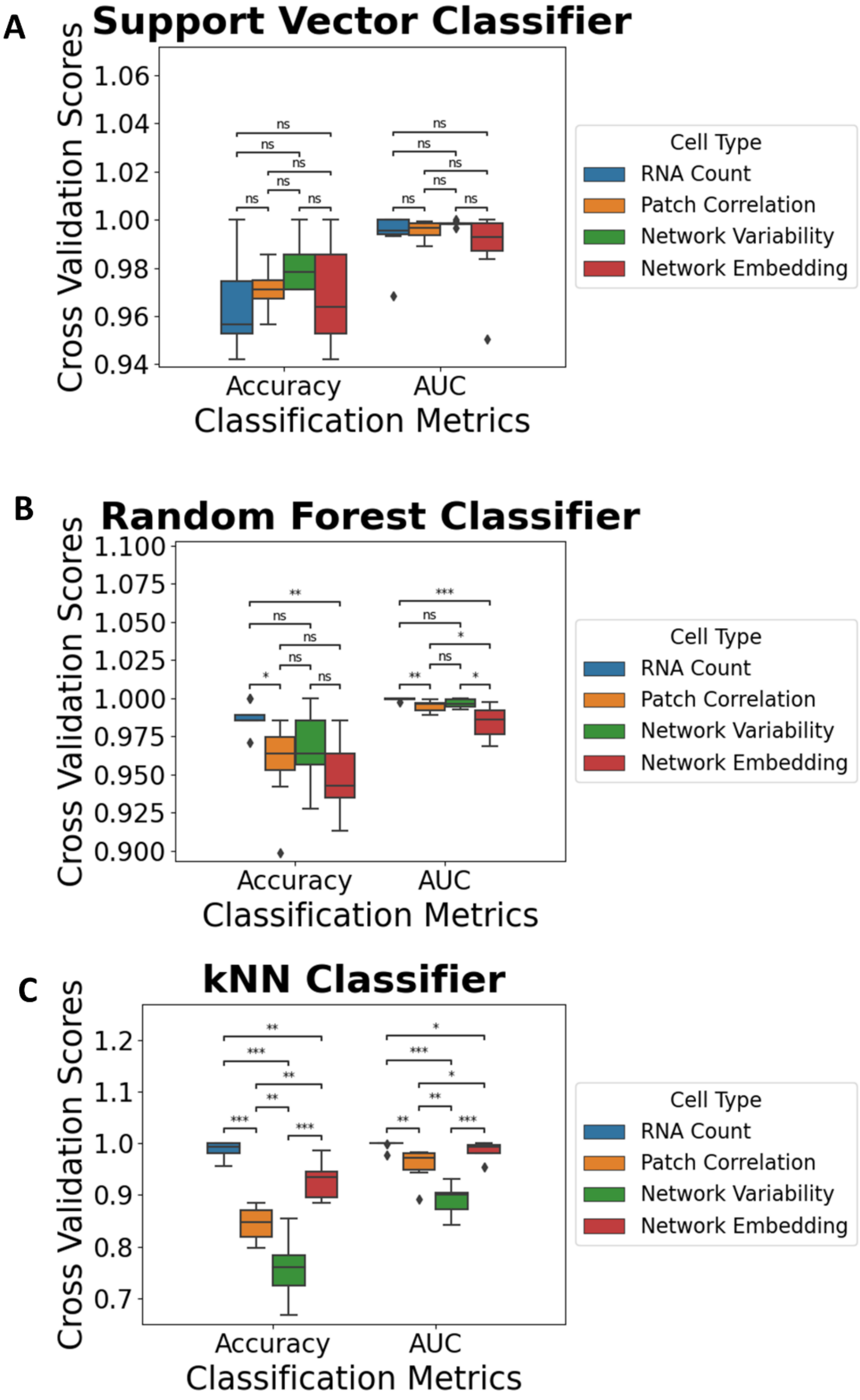
A
Support Vector Classifier
B
Random Forest Classifier
C
kNN Classifier
Cross Validation Scores
Classification Metrics
Accuracy
AUC
Cell Type
RNA Count
Patch Correlation
Network Variability
Network Embedding

Fig. S10: Classification of BM-MSCs v. UC-MSCs using multiple classifiers

A. Classification performance of support vector machine. The statistical comparison was generated using 5-fold cross-validation. The data were shuffled and divided into five folds. A classification algorithm was trained with each fold of the data used as the testing set and the remaining data as the training set. Classification using a support vector classifier does not significantly differ between single-cell RNA count, patch correlation, network variability, and autoencoder encoding.
B. Classification performance of random forest. The classification performance is ranked from the best to the worst single-cell RNA count, single-cell network variability, patch correlation, and autoencoder encoding.
C. Classification performance of k nearest neighbor (kNN) classifier. The classification performance is ranked from the best to the worst single-cell RNA count, autoencoder encoding, patch correlation, and single-cell network variability.

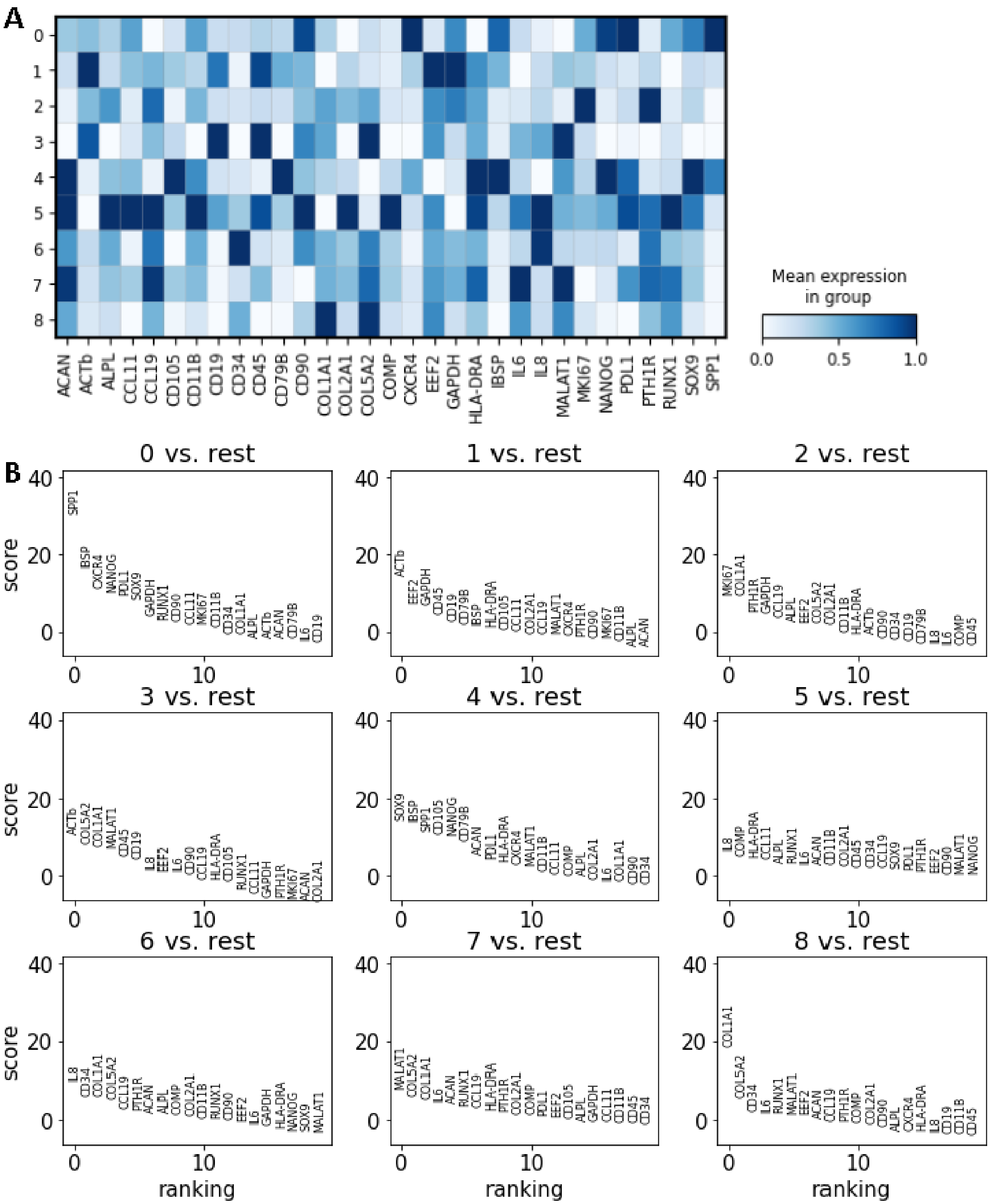

A
B
Mean expression
in group
0.0
0.5
1.0
ACAN
ACTb
ALPL
CCL11
CCL19
CD105
CD11B
CD19
CD34
CD45
CD79B
CD90
COL1A1
COL2A1
COL5A2
COMP
CXCR4
EEF2
GAPDH
HLA-DRA
IBSP
IL6
IL8
MALAT1
MKI67
NANOG
PDL1
PTH1R
RUNX1
SOX9
SPP1
0 vs. rest
1 vs. rest
2 vs. rest
3 vs. rest
4 vs. rest
5 vs. rest
6 vs. rest
7 vs. rest
8 vs. rest
score
ranking

Fig. S11: Clustering of MSCs based on single-cell RNA count

A. Mean expression of the nine identified clusters of MSCs based on single-cell RNA count.
B. High-rank genes of each cluster. The gene ranking algorithm identifies the marker gene of each cluster. The clustering identifies several subtypes of MSCs. For example, cluster 0 shows elevated osteogenic markers SPP1 and IBSP, and cluster 8 shows elevated extracellular matrix genes such as COL1A1 and COL5A2. The identified subtypes of MSCs were used to define homotypic and heterotypic CCC.

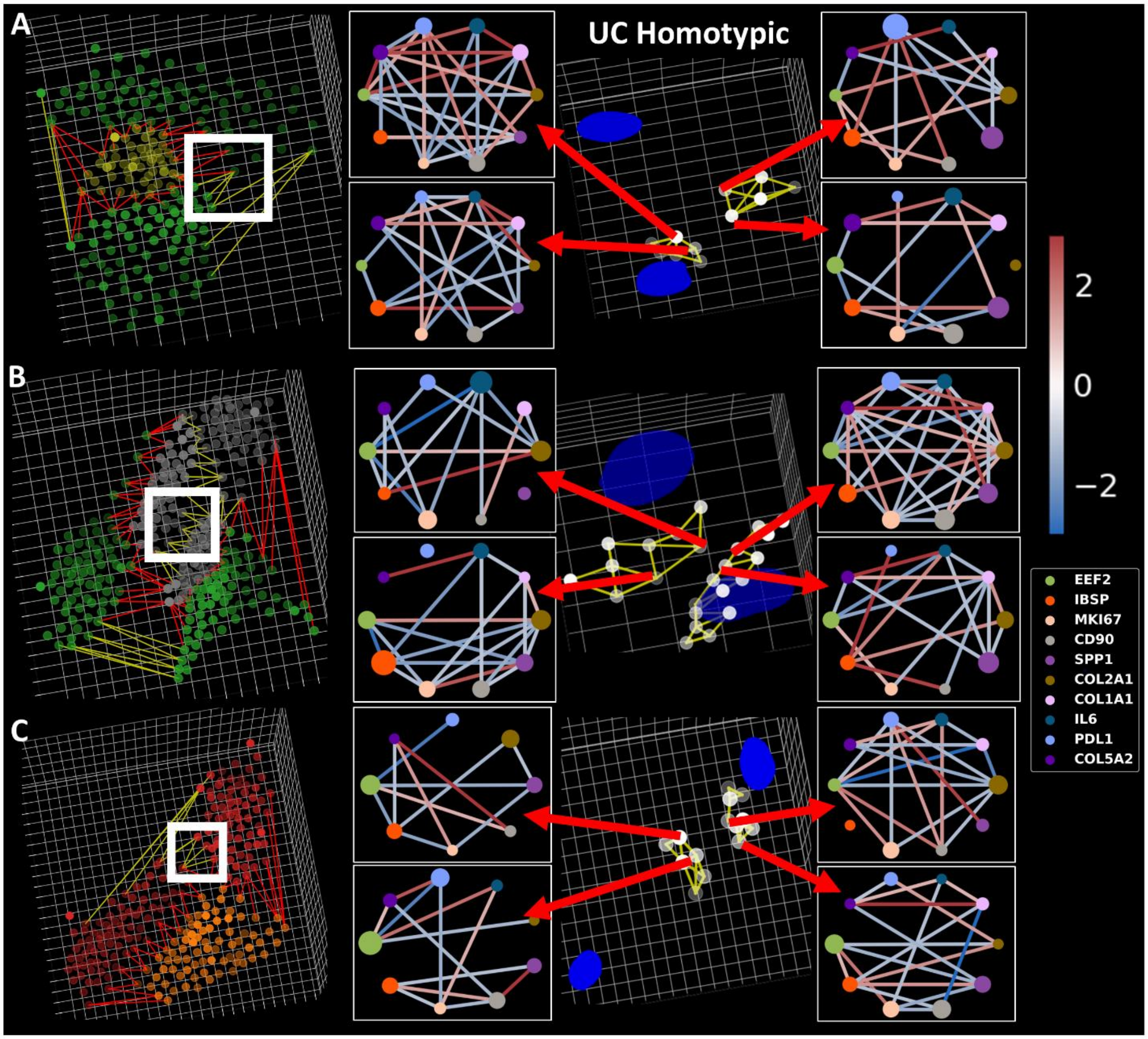

Fig. S12: Examples of homotypic CCC between UC-MSCs.

The local gene neighborhood networks of homotypic patches of UC-MSCs are shown in this figure. The x and y axes have grid spacing of 10µm and the z-axis has grid spacing of 1µm.

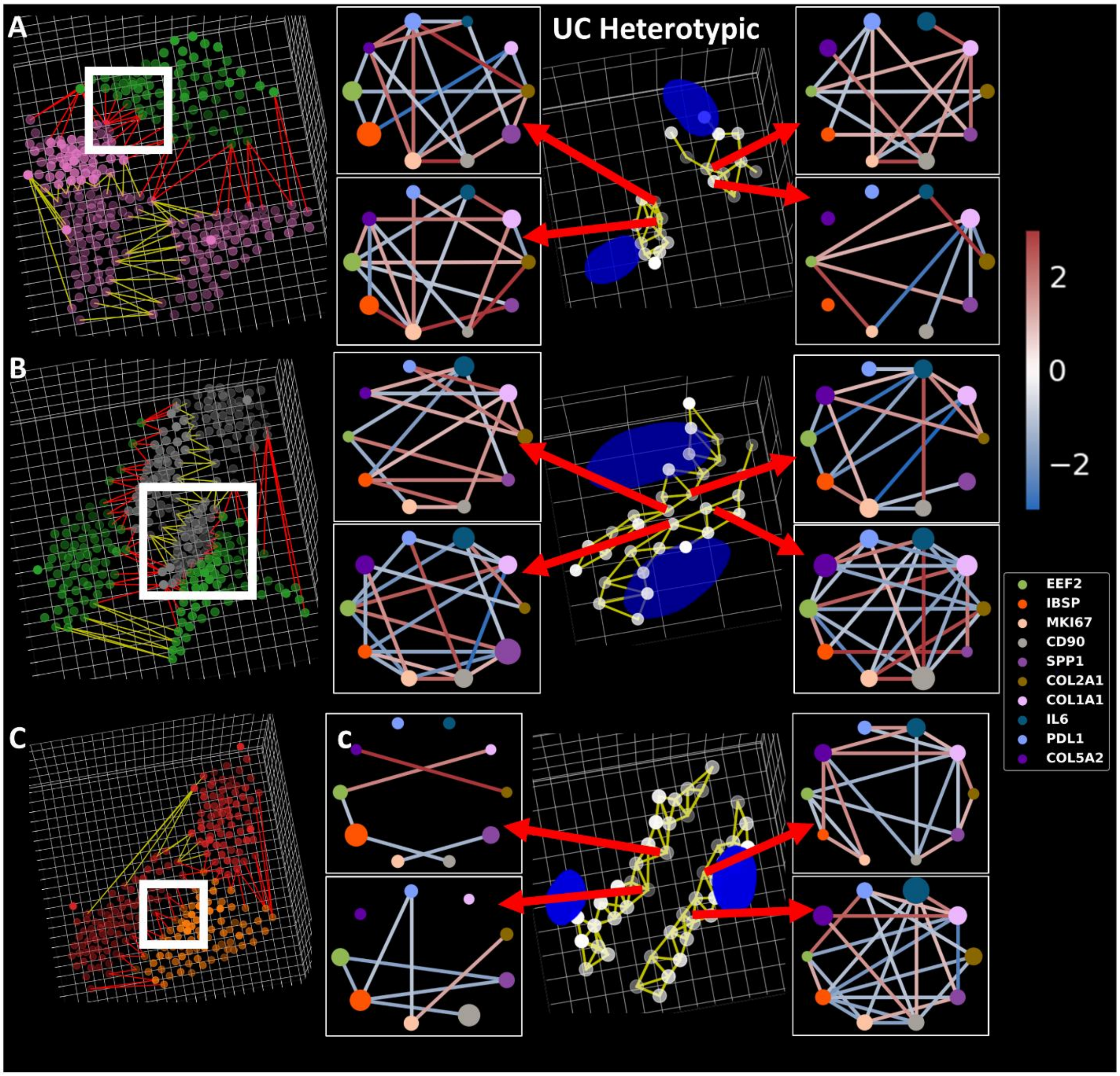

Fig. S13: Examples of heterotypic CCC between UC-MSCs.

The local gene neighborhood networks of heterotypic patches of UC-MSCs are shown in this figure. The x and y axes have grid spacing of 10µm and the z-axis has grid spacing of 1µm.

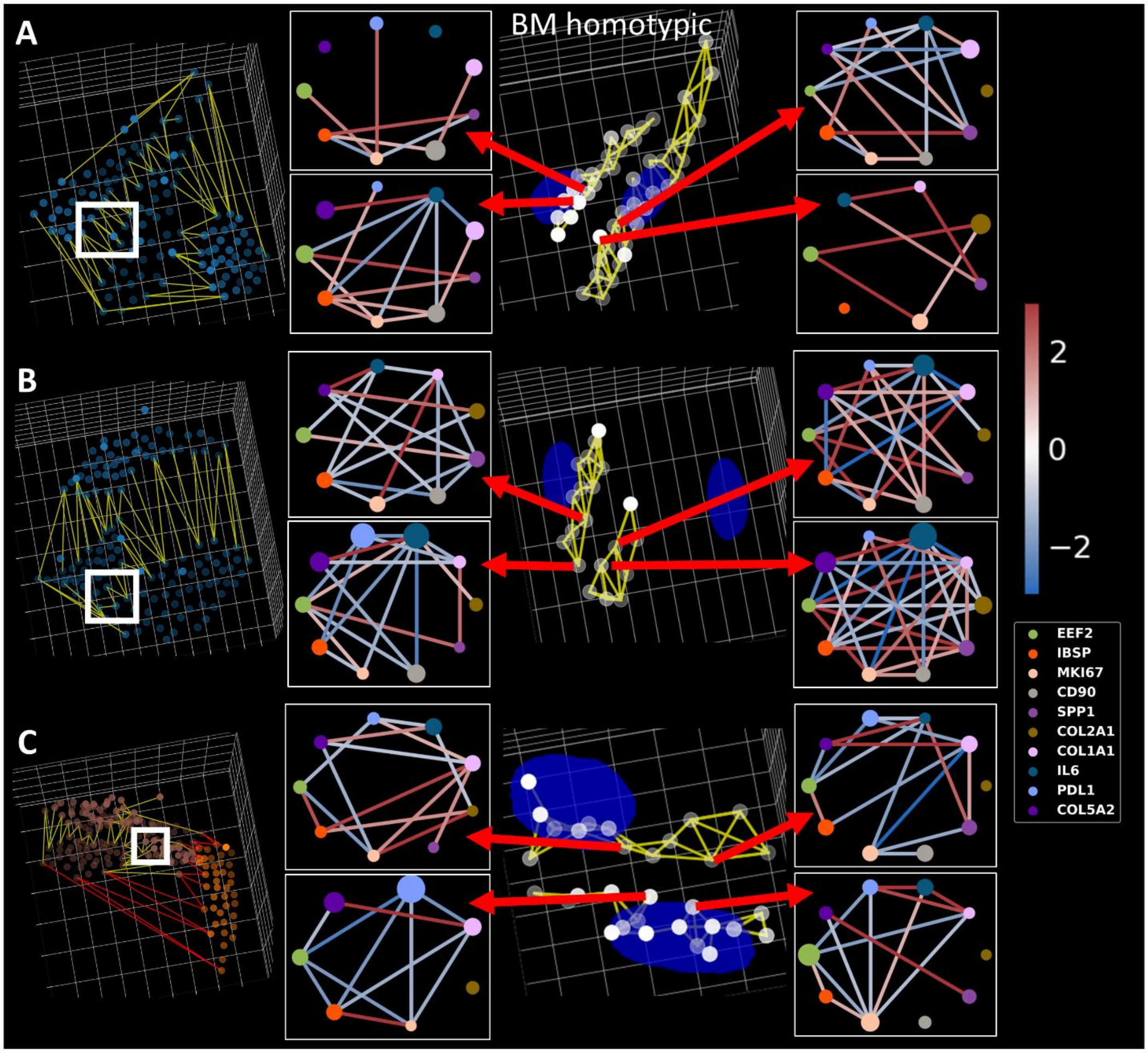

Fig. S14: Examples of homotypic CCC between BM-MSCs.

The local gene neighborhood networks of homotypic patches of BM-MSCs are shown in this figure. The x and y axes have grid spacing of 10μm and the z-axis has grid spacing of 1μm.

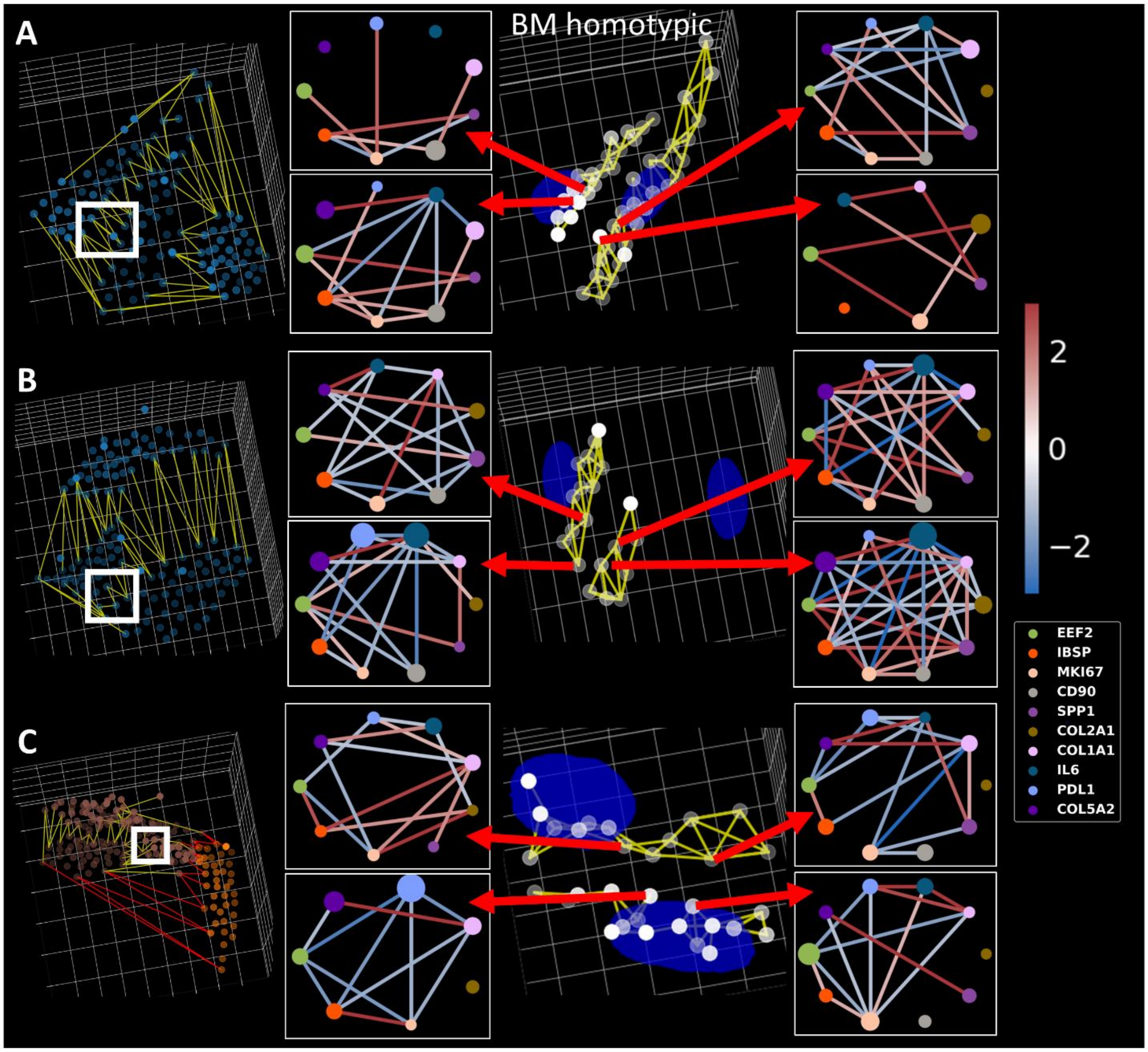

Fig. S15: Examples of heterotypic interactions between BM-MSCs.

The local gene neighborhood networks of homotypic patches of BM-MSCs are shown in this figure. The x and y axes have grid spacing of 10µm. The z-axis grid spacing is 1µm in A and C and 5µm in B.

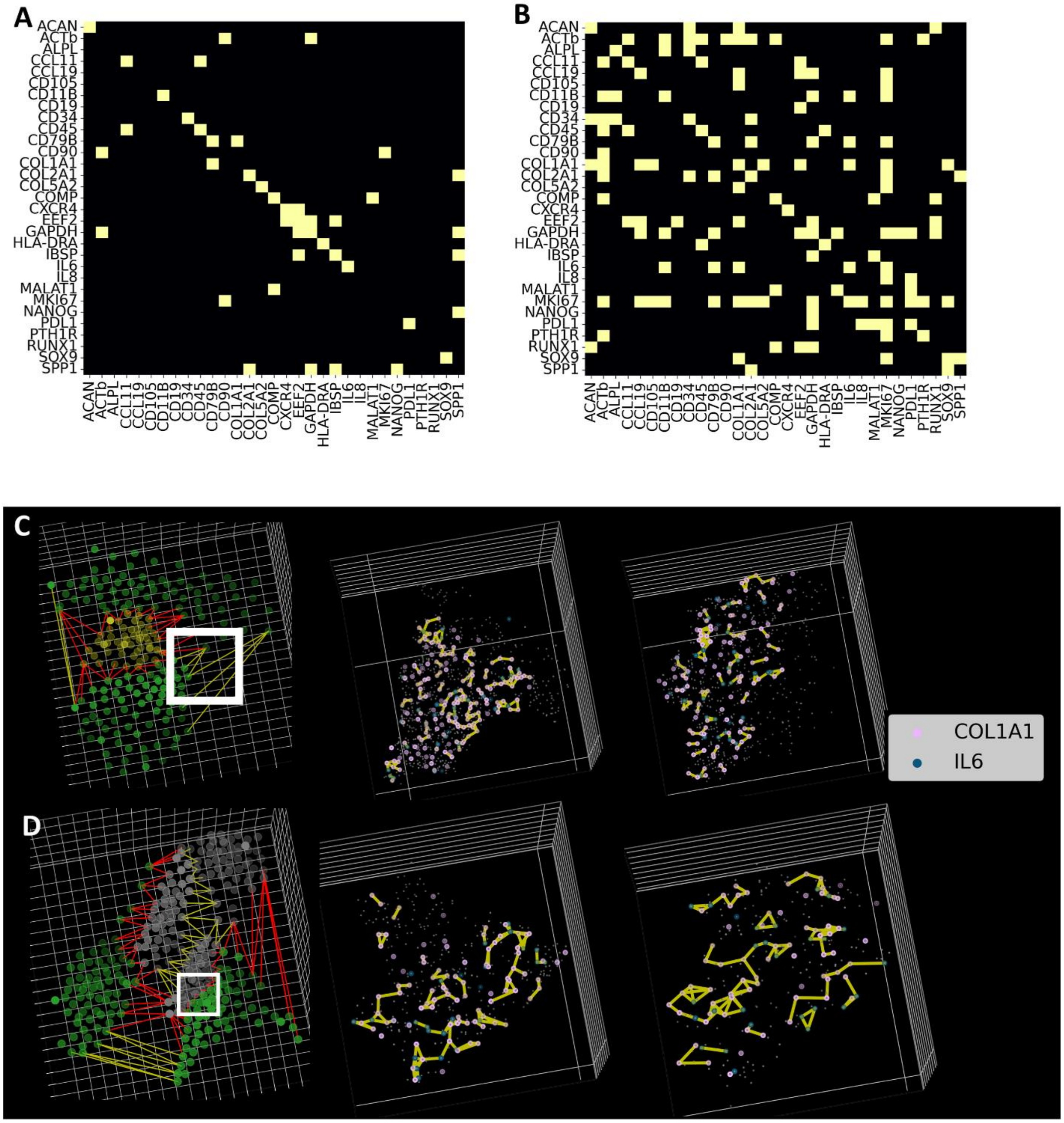
A
B
C
D
ACAN
ACTb
ALPL
CCL11
CCL19
CD105
CD11B
CD19
CD34
CD45
CD79B
CD90
COL1A1
COL2A1
COL5A2
COMP
CXCR4
EEF2
GAPDH
HLA-DRA
IBSP
IL6
IL8
MALAT1
MKI67
NANOG
PDL1
PTH1R
RUNX1
SOX9
SPP1
COL1A1
IL6

Fig. S16: Comparison of gene proximity in border patches

A. Statistical significance between pairwise gene proximity scores of homotypic and heterotypic border patches between BM-MSCs. The highlighted gene-pairs show statistically significant differences between homotypic and heterotypic border patches with α=0.05.

B. Statistical significance between pairwise gene proximity scores of homotypic and heterotypic border patches between UC-MSCs.

C. Boxplot comparing proximity scores of border patches between BM-MSCs. Homotypic border patches show higher EEF2-IBSP and lower COMP-MKI67 proximity. The statistical significance annotation is as follow: p-value annotation legend: ns: 5.00e-02 < p <= 1.00e+00, *: 1.00e-02 < p <= 5.00e-02, **: 1.00e-03 < p <= 1.00e-02, ***: 1.00e-04 < p <= 1.00e-03, ****: p <= 1.00e-04

D. Boxplot comparing proximity scores of border patches between UC-MSCs. Homotypic border patches show higher COL1A1-IL6, MKI67-PDL1, and COL1A1-COL5A2 proximity.

E. COL1A1-IL6 neighborhoods of homotypic border patches between UC-MSCs. The two homotypic patches from two UC-MSCs highlighted in the left panel are shown. Each scatter is a detected transcript, and the COL1A1 and IL6 transcripts are highlighted according to the color legend. The yellow edges shows the detected neighbors between COL1A1 and IL6 transcripts.

F. COL1A1-IL6 neighborhoods of heterotypic border patches between UC-MSCs. The x and y axes have grid spacing of 10μm and the z-axis has grid spacing of 1μm.

A

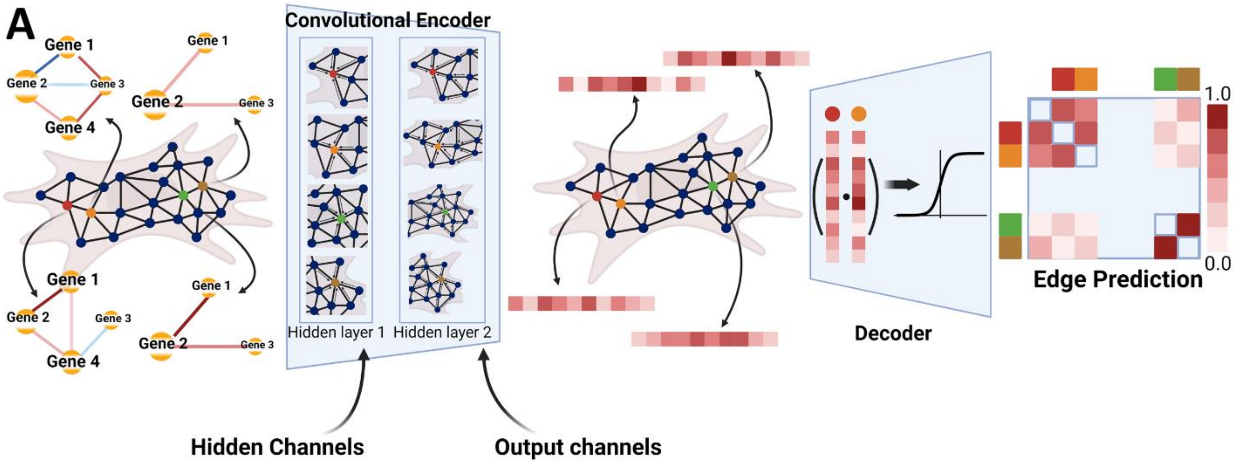

Convolutional Encoder
Gene 1
Gene 2
Gene 3
Gene 4
Gene 1
Gene 2
Gene 3
Gene 1
Gene 2
Gene 3
Gene 4
Gene 1
Gene 2
Gene 3
Hidden layer 1
Hidden layer 2
Hidden Channels
Output channels
Decoder
1.0
0.0
Edge Prediction

B

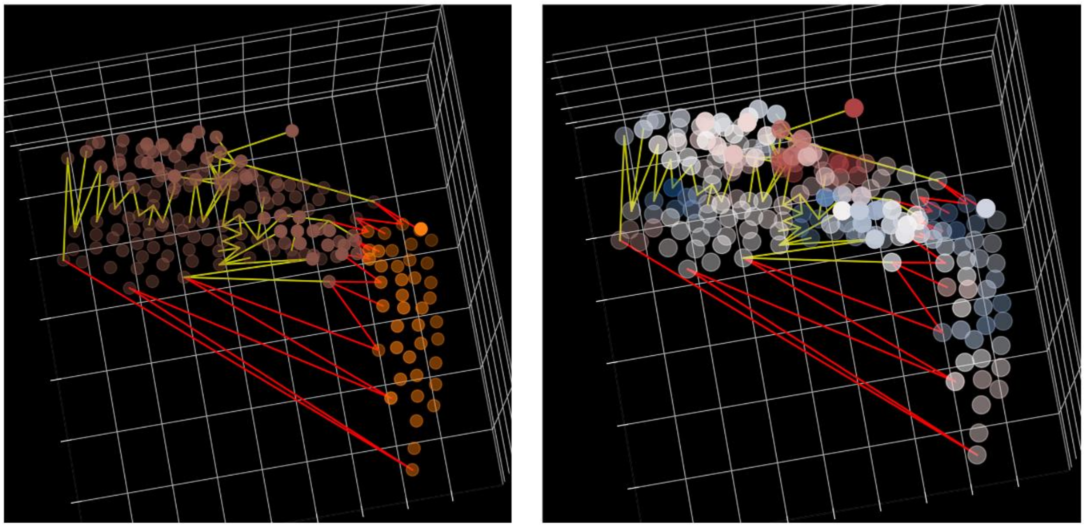

Fig. S17: Autoencoder embedding of border patches

A. Autoencoder architecture. The autoencoder consists of a convolutional encoder and an inner product decoder. The convolutional encoder uses two graph convolutional layers to encode the gene proximity into features in latent space. The inner product decoder uses the latent space features to predict edges between each pair of nodes

B. CCC patches and embedding values of subcellular patches. The homotypic and heterotypic patches are connected by yellow and red edges (left). The nodes were also colored by an embedded feature in the latent space (right). The red node color indicates larger values, and blue indicates smaller values. The figure shows the variation of embedding value between heterotypic and homotypic patches. The x and y axes have grid spacing of 10µm and the z-axis has grid spacing of 1µm.

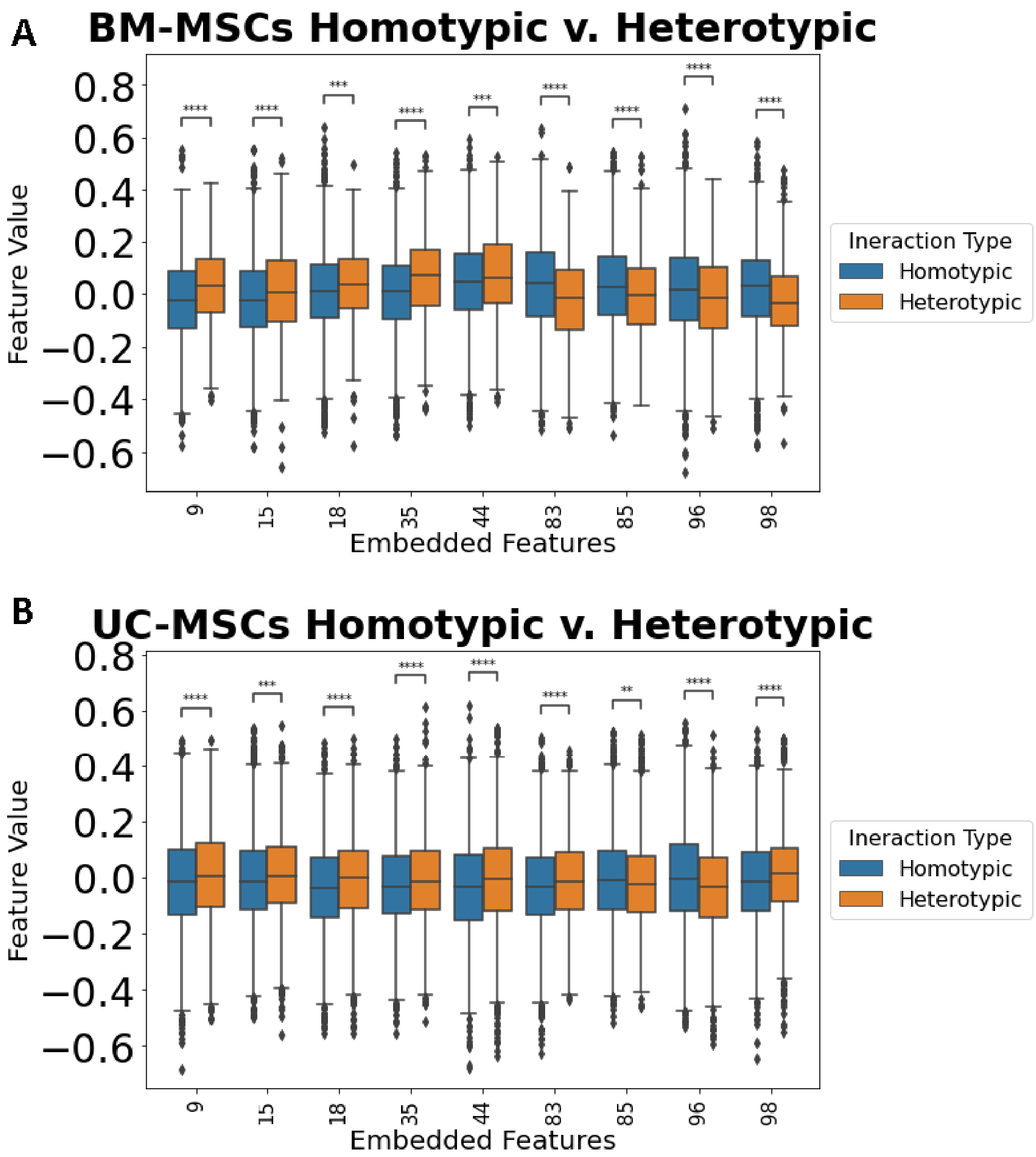

Fig. S18: Comparison of embedding values between homotypic and heterotypic patches

A. Comparison of embedding values of BM-MSC border patches.
B. Comparison of embedding values of UC-MSC border patches.

The statistical annotation follows the legend: ns: 5.00e-02 < p <= 1.00e+00, *: 1.00e-02 < p <= 5.00e-02, **: 1.00e-03 < p <= 1.00e-02, ***: 1.00e-04 < p <= 1.00e-03, ****: p <= 1.00e-04.

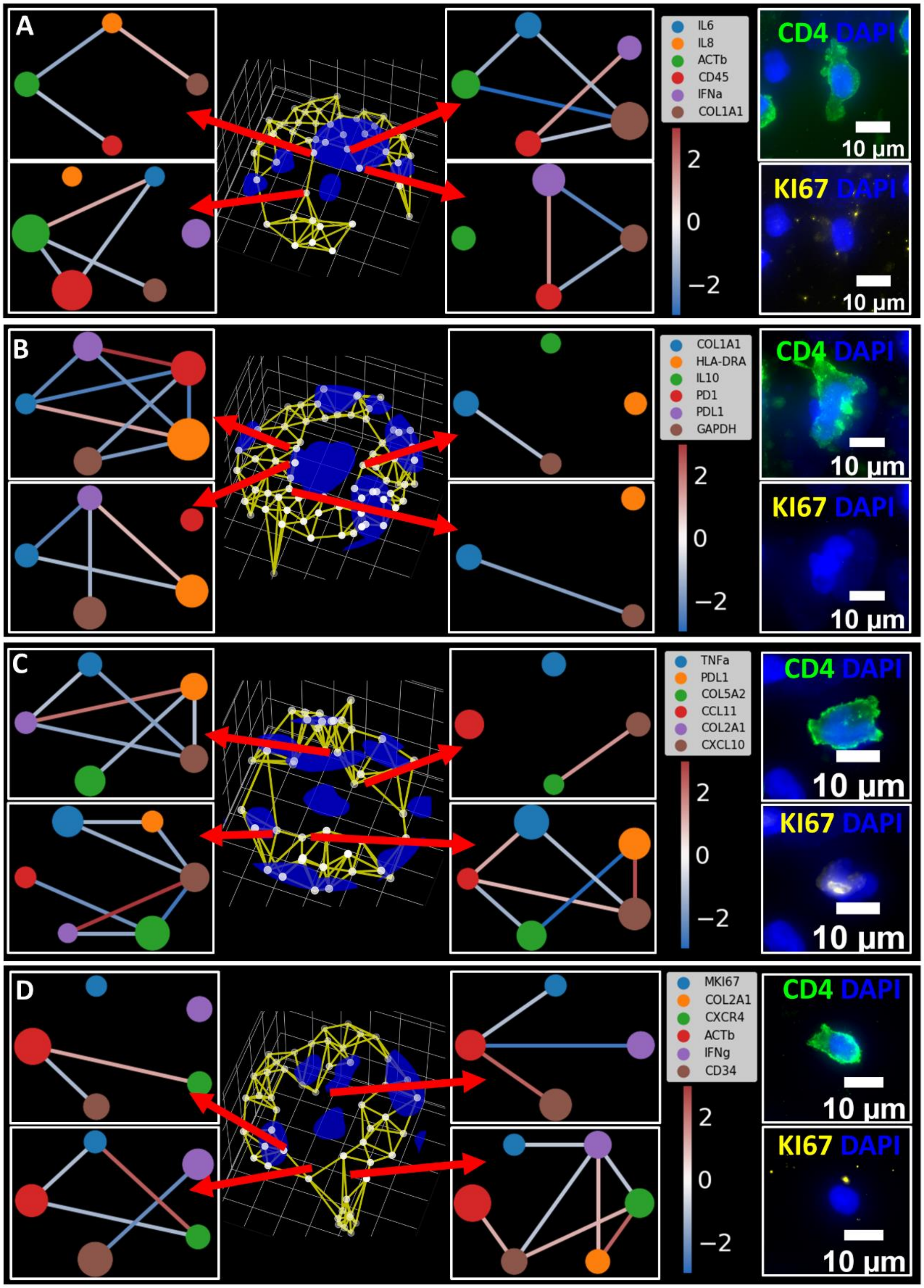

A
IL6
IL8
ACTb
CD45
IFNa
COL1A1
2
0
−2
CD4 DAPI
10 µm
KI67 DAPI
10 µm
B
COL1A1
HLA-DRA
IL10
PD1
PDL1
GAPDH
2
0
−2
CD4 DAPI
10 µm
KI67 DAPI
10 µm
C
TNFa
PDL1
COL5A2
CCL11
COL2A1
CXCL10
2
0
−2
CD4 DAPI
10 µm
KI67 DAPI
10 µm
D
MKI67
COL2A1
CXCR4
ACTb
IFNg
CD34
2
0
−2
CD4 DAPI
10 µm
KI67 DAPI
10 µm

Fig. S19: Examples of transcriptional microenvironments around CD4+ t-cells.

The four panels show four different examples of transcriptional microenvironments around CD4+ t-cells. The patch nearest neighbor graph is shown by scatter plot of patch center positions and edges of the graph shown by yellow edges. The gene neighborhood network of the patches near the immune cell is shown. Each gene neighborhood graph shows the genes and their proximity. The IF staining labeled CD4 and KI67, indicating proliferating and nonproliferating CD4+ t-cells. The grid spacing of all axes is 10μm.

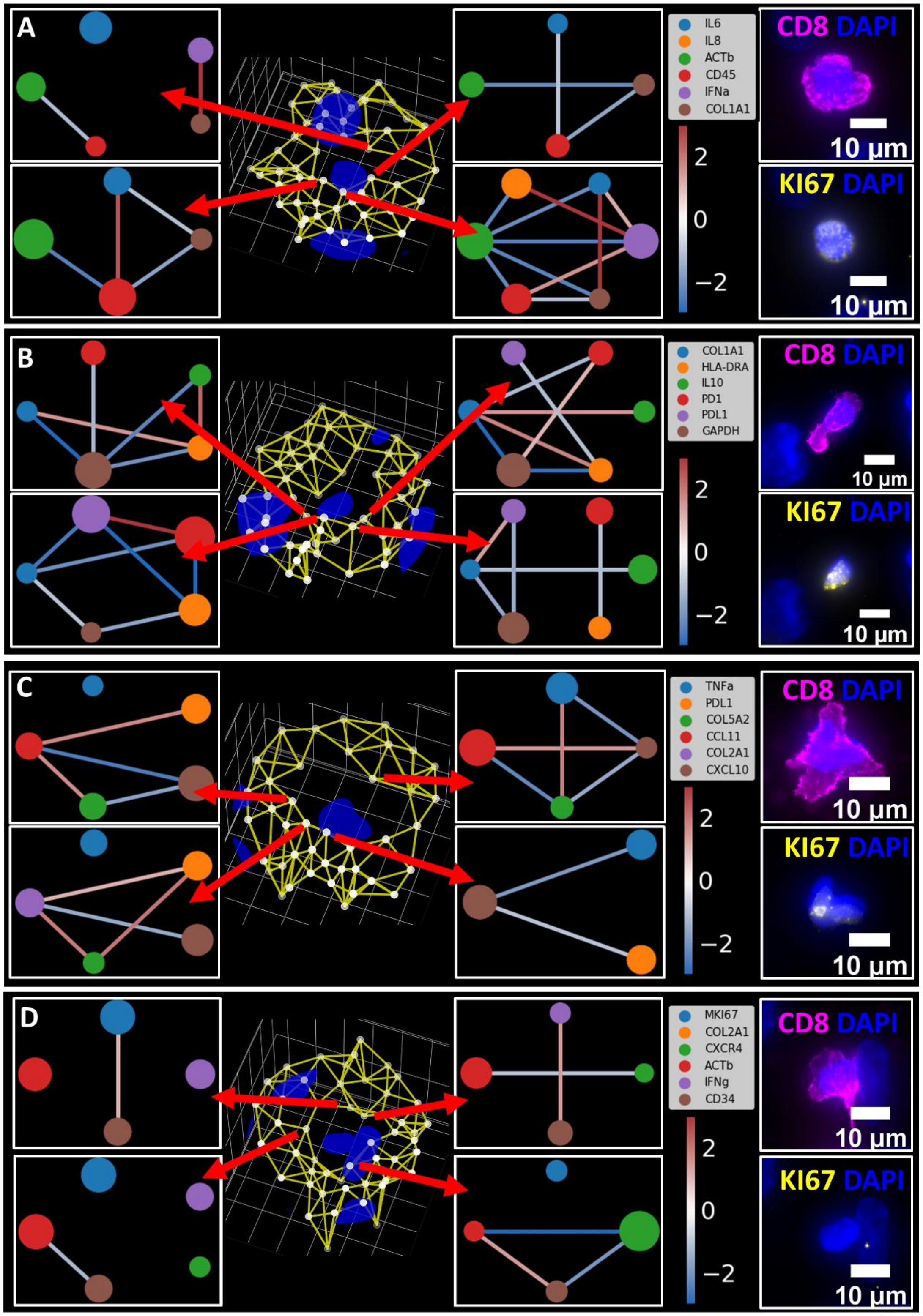

A
IL6
IL8
ACTb
CD45
IFNa
COL1A1
2
0
−2
CD8 DAPI
10 μm
KI67 DAPI
10 μm
B
COL1A1
HLA-DRA
IL10
PD1
PDL1
GAPDH
2
0
−2
CD8 DAPI
10 μm
KI67 DAPI
10 μm
C
TNFa
PDL1
COL5A2
CCL11
COL2A1
CXCL10
2
0
−2
CD8 DAPI
10 μm
KI67 DAPI
10 μm
D
MKI67
COL2A1
CXCR4
ACTb
IFNg
CD34
2
0
−2
CD8 DAPI
10 μm
KI67 DAPI
10 μm

Fig. S20: Examples of transcriptional microenvironments around CD4+ t-cells

The four panels show four different examples of transcriptional microenvironments around CD8+ t-cells. The patch nearest neighbor graph is shown by a scatter plot of patch center positions and edges of the graph shown by yellow edges. The gene neighborhood network of the patches near the immune cell is shown. Each gene neighborhood graph shows the genes and their proximity. The IF staining labeled CD8 and KI67, indicated proliferating and nonproliferating CD8+ t-cells. The grid spacing of all axes is 10µm.

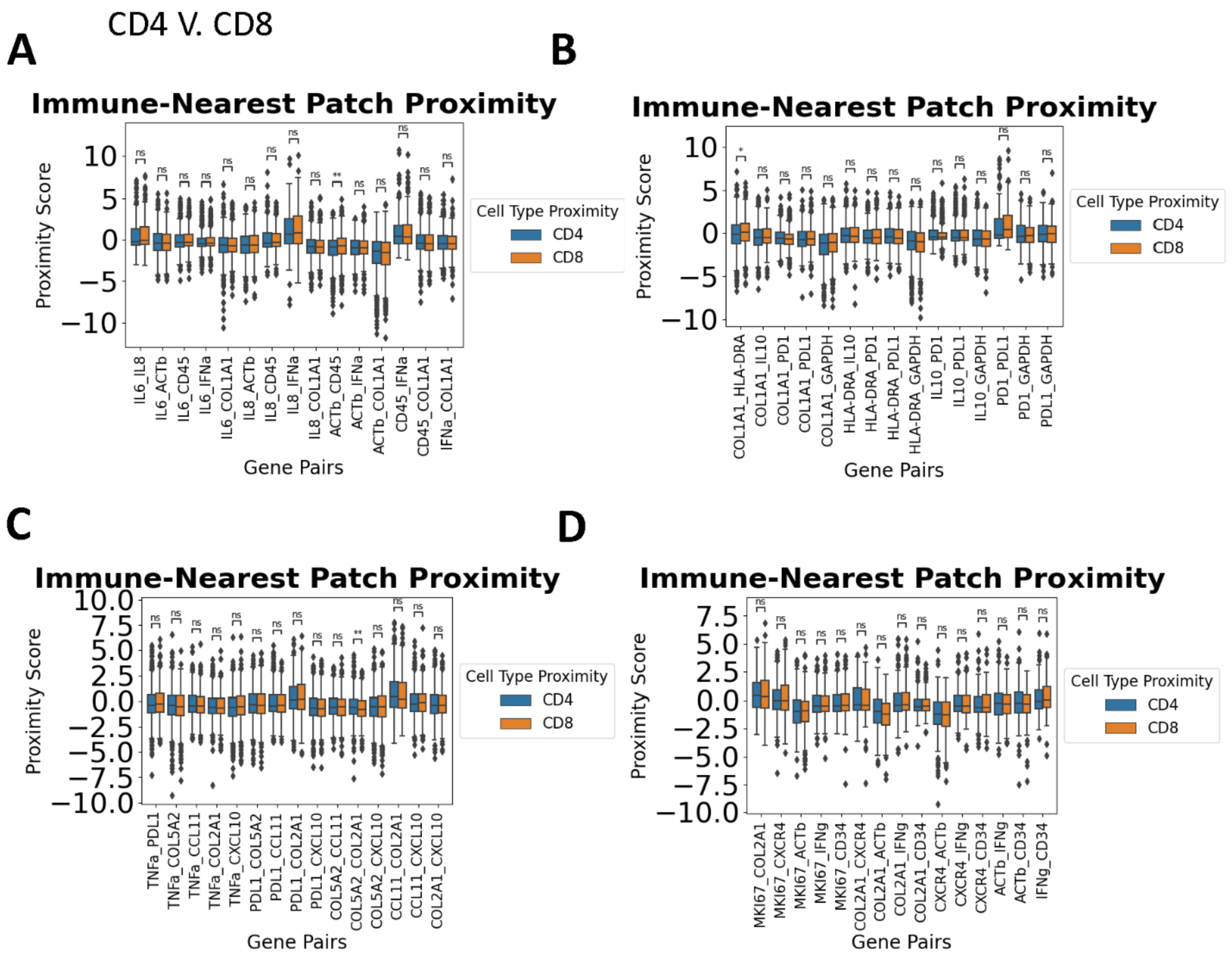

Fig. S21: Gene proximity comparison of patches nearest to CD4+ t-cells and CD8+ t-cells.

Four sets of multiplexed RNA-FISH experiments were performed to expand the number of genes studied in the MSC-PBMC co-culture samples. The five patches in the transcriptional microenvironment nearest to the central immune cells were compared. The patches were labeled according to the cell types of the central immune cell as CD4+ or CD8+ t-cells. Each panel in this figure represents the gene proximity comparison for each of the four sets of experiments. Patches near CD8+ t-cells show higher ACTb-CD45, COL1A1-HLA-DRA, and lower COL5A2-COL2A1 proximity.

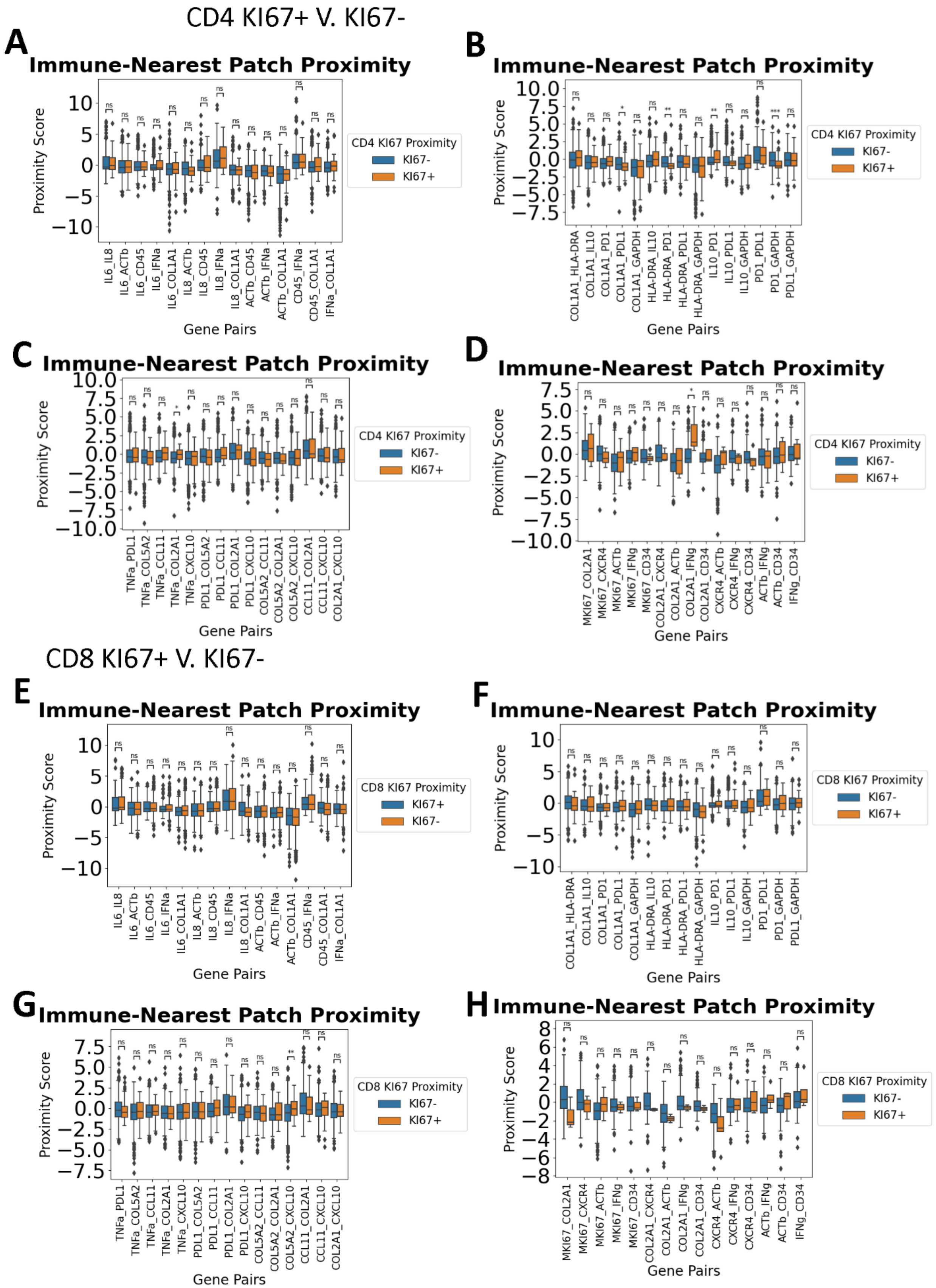

CD4 KI67+ V. KI67-
A
Immune-Nearest Patch Proximity
Proximity Score
Gene Pairs
IL6_IL8
IL6_ACTb
IL6_CD45
IL6_IFNa
IL6_COL1A1
IL8_ACTb
IL8_CD45
IL8_IFNa
IL8_COL1A1
ACTb_CD45
ACTb_IFNa
ACTb_COL1A1
CD45_IFNa
CD45_COL1A1
IFNa_COL1A1
CD4 KI67 Proximity
KI67-
KI67+
B
Immune-Nearest Patch Proximity
Proximity Score
Gene Pairs
COL1A1_HLA-DRA
COL1A1_IL10
COL1A1_PD1
COL1A1_PDL1
COL1A1_GAPDH
HLA-DRA_IL10
HLA-DRA_PD1
HLA-DRA_PDL1
HLA-DRA_GAPDH
IL10_PD1
IL10_PDL1
IL10_GAPDH
PD1_PDL1
PD1_GAPDH
PDL1_GAPDH
CD4 KI67 Proximity
KI67-
KI67+
C
Immune-Nearest Patch Proximity
Proximity Score
Gene Pairs
TNFa_PDL1
TNFa_COL5A2
TNFa_CCL11
TNFa_COL2A1
TNFa_CXCL10
PDL1_COL5A2
PDL1_CCL11
PDL1_COL2A1
PDL1_CXCL10
COL5A2_CCL11
COL5A2_COL2A1
COL5A2_CXCL10
CCL11_COL2A1
CCL11_CXCL10
COL2A1_CXCL10
CD4 KI67 Proximity
KI67-
KI67+
D
Immune-Nearest Patch Proximity
Proximity Score
Gene Pairs
MKI67_COL2A1
MKI67_CXCR4
MKI67_ACTb
MKI67_IFNg
MKI67_CD34
COL2A1_CXCR4
COL2A1_ACTb
COL2A1_IFNg
COL2A1_CD34
CXCR4_ACTb
CXCR4_IFNg
CXCR4_CD34
ACTb_IFNg
ACTb_CD34
IFNg_CD34
CD4 KI67 Proximity
KI67-
KI67+
CD8 KI67+ V. KI67-
E
Immune-Nearest Patch Proximity
Proximity Score
Gene Pairs
CD8 KI67 Proximity
KI67+
KI67-
F
Immune-Nearest Patch Proximity
Proximity Score
Gene Pairs
CD8 KI67 Proximity
KI67-
KI67+
G
Immune-Nearest Patch Proximity
Proximity Score
Gene Pairs
CD8 KI67 Proximity
KI67-
KI67+
H
Immune-Nearest Patch Proximity
Proximity Score
Gene Pairs
CD8 KI67 Proximity
KI67-
KI67+

Fig. S22: Gene proximity comparison between patches nearest to KI67+ t-cells and KI67- t-cells.

A, B, C, D. Gene proximity comparison between patches contacting KI67+ and KI67- and CD4+ t-cells. The five patches in the transcriptional microenvironment nearest to the central immune cells were compared. Among CD4+ t-cells, patches near KI67+ cells show higher IL10-PD1, TNFa-COL2A1, and COL2A1-IFNg proximity, and lower COL1A1-PDL1, HLA-DRA-PD1, and PD1-GAPDH proximity.

E, F, G, H. Gene proximity comparison between patches contacting KI67+ and KI67- and CD8+ t-cells. Among CD8+ t-cells, patches near KI67+ cells show higher COL5A2-CXCL10 proximity.

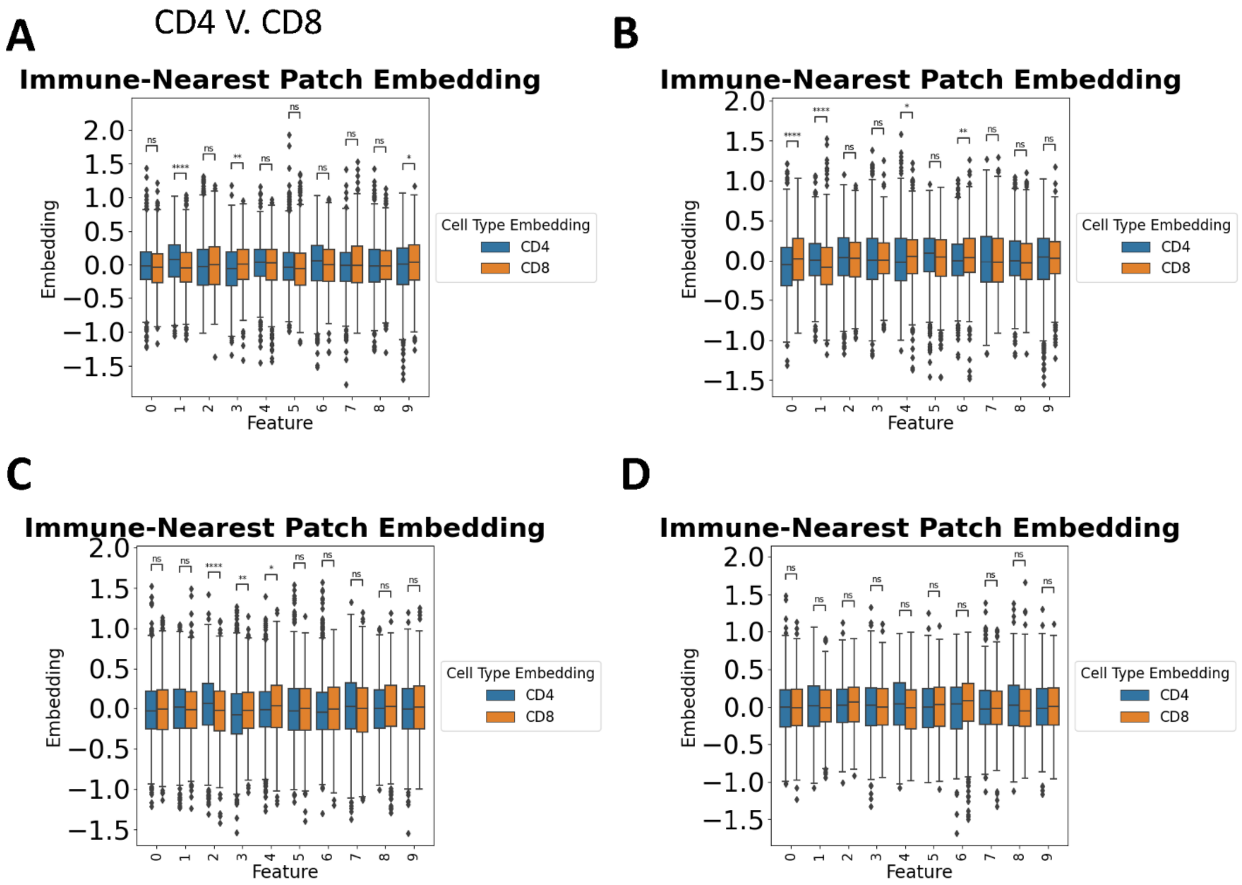

Fig. S23: Autoencoder embedding comparison of patches nearest to CD4+ t-cells and CD8+ t-cells.

The autoencoder embedding value comparison of the immune-nearest patch of transcriptional microenvironment around CD4+ and CD8+ t-cells. The embedding increases the number of significantly different features when compared to gene proximity of the same patches.

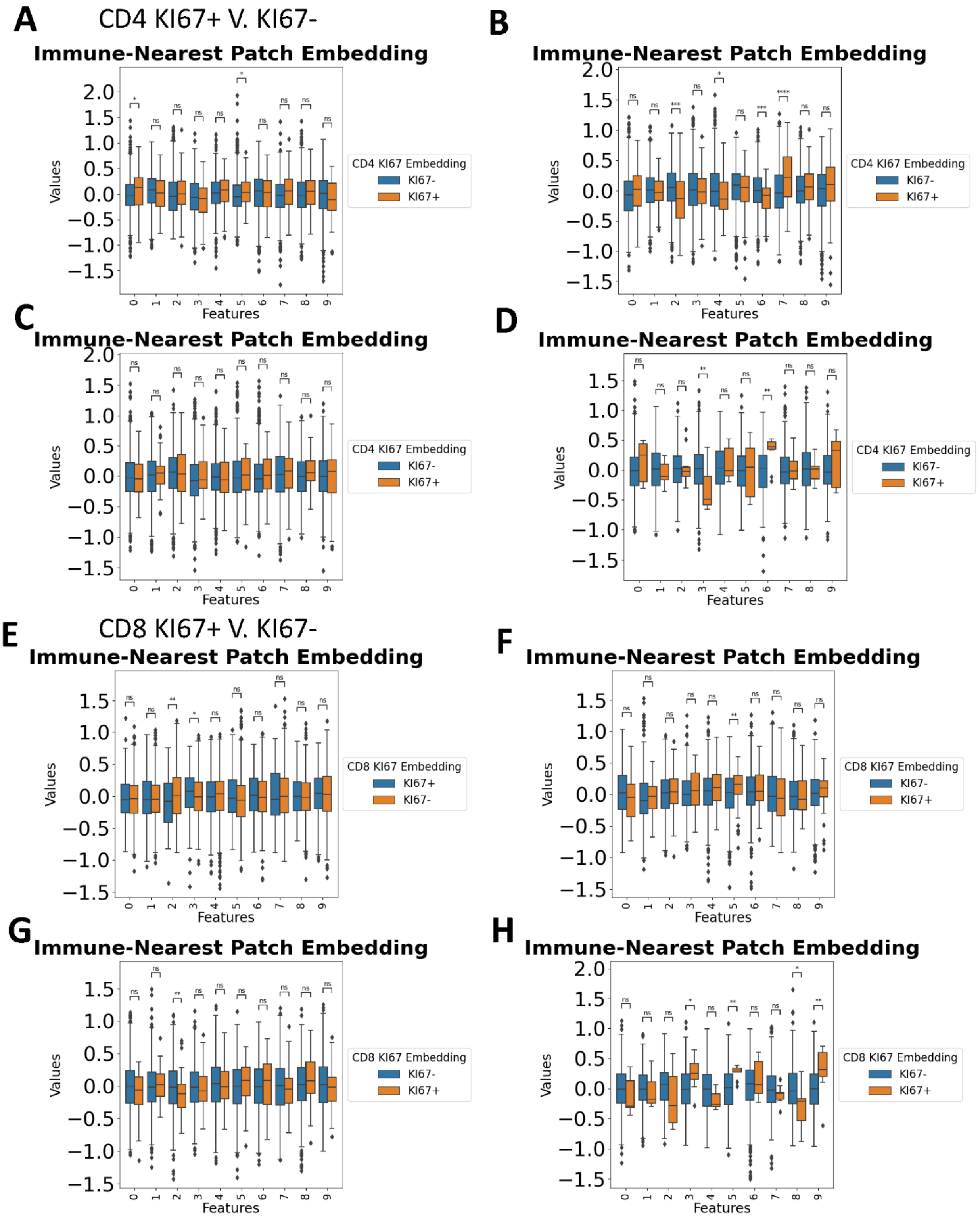
A
CD4 KI67+ V. KI67-
Immune-Nearest Patch Embedding
Values
Features
CD4 KI67 Embedding
KI67-
KI67+
B
Immune-Nearest Patch Embedding
C
Immune-Nearest Patch Embedding
D
Immune-Nearest Patch Embedding
E
CD8 KI67+ V. KI67-
Immune-Nearest Patch Embedding
CD8 KI67 Embedding
KI67+
KI67-
F
Immune-Nearest Patch Embedding
G
Immune-Nearest Patch Embedding
H
Immune-Nearest Patch Embedding

Fig. 24: Autoencoder embedding comparison between patches contacting KI67+ t-cells and KI67- t-cells.

A, B, C, D. Autoencoder embedding comparison between patches contacting KI67+ and KI67- and CD4+ t-cells.

E, F, G, H. Autoencoder embedding comparison between patches contacting KI67+ and KI67- and CD8+ t-cells. Both comparisons among CD4+ t-cells and CD8+ t-cells of embedding features show more features to be statistically significant.

Experiment set 1	IL8	Experiment set 2	HLA-DRA
	IL6		COL1A1
	ACTb		IL10
	COL1A1		PDL1
	IFNa		GAPDH
	CD45		PD1
Experiment set 3	COL5A2	Experiment set 4	COL2A1
	TNFa		MKI67
	PDL1		CXCR4
	COL2A1		ACTb
	CXCL10		CD34
	CCL11		IFNg

Supplementary Table 1: Genes for multiplexed HCR-FISH in MSC-PBMC coculture system.
